# Electrocorticographic characteristics of rats exposed to hyperbaric nitrox

**DOI:** 10.3389/fphys.2026.1976565

**Published:** 2026-10-21

**Authors:** Chenglong Li, Shuo Yan, Rihui Shen, Haoze Zheng, Kun Wang, Jinjun Tang, Wenwu Liu, Shifeng Wang

**Affiliations:** 1 Naval Medical Center of the People’s Liberation Army (PLA), Naval Medical University, Shanghai, China; 2 School of Medicine, Shanghai University, Shanghai, China

**Keywords:** electrocorticographic characteristics, electrocorticography, frequency-band power, hyperbaric exposure, hyperbaric nitrox

## Abstract

**Background:**

This study aimed to characterize and compare the dynamic changes in cortical electrophysiological activity during hyperbaric nitrox exposure and subsequent hyperbaric heliox exposure in rats receiving continuous electrocorticographic (ECoG) recording.

**Methods:**

Male rats were randomly assigned to the 3% O_2_–N_2_, 16% O_2_–N_2_, and 100% O_2_ groups (n=8 per group). Rats in the 3% O_2_–N_2_ and 16% O_2_–N_2_ groups were exposed to their respective gas mixtures at 700 kPa for 60 min, whereas those in the 100% O_2_ group were exposed to pure oxygen at 200 kPa for 60 min. Following hyperbaric nitrox exposure, the 16% O_2_–N_2_ mixture was replaced with a 16% O_2_–84% He mixture at the same pressure for additional 60 min. ECoG was continuously recorded, and power spectral density (PSD), absolute power in the δ, θ, α, β, and γ bands, delta–theta/alpha–beta ratio (DTABR), median frequency (MF), sample entropy (SampEn), and Higuchi fractal dimension (HFD) were calculated and compared among groups.

**Results:**

Rats in the 3% O_2_–N_2_ and 16% O_2_–N_2_ groups exhibited increased slow-wave activity and a shift in spectral power toward lower frequencies, accompanied by increased δ-, θ-, α-, and β-band power, decreased γ-band power, increased DTABR, and reduced MF, SampEn, and HFD as compared to the baseline levels. Changes in several ECoG parameters emerged earlier in the 3% O_2_–N_2_ group than in the 16% O_2_–N_2_ group. Compared with the 100% O_2_ group, rats in the 3% O_2_–N_2_ and 16% O_2_–N_2_ groups generally exhibited lower MF, SampEn, and HFD, whereas ECoG parameters in the 100% O_2_ group showed relatively minor changes as compared to the baseline levels. Following substitution with hyperbaric heliox, DTABR tended to decrease, whereas MF, SampEn, and HFD tended to increase, suggesting a trend toward baseline ECoG characteristics.

**Conclusions:**

Hyperbaric nitrox exposure was associated with a shift in ECoG activity toward lower frequencies, reduced signal complexity, and greater regularity in rats. Following substitution with hyperbaric heliox, these ECoG characteristics showed trends toward baseline levels. These findings characterize cortical electrophysiological alterations associated with hyperbaric nitrox exposure and may provide a basis for future investigations into the electrophysiological characteristics of nitrogen narcosis.

## 1 Introduction

Nitrox is a breathing gas mixture composed of nitrogen and oxygen. As diving depth increases, the partial pressure of nitrogen (PN_2_) in the breathing mixture increases with increasing ambient pressure. Hyperbaric nitrox exposure may induce nitrogen narcosis (Marienau and Arguin, 2013), which is characterized by impaired cognition, judgment, and motor coordination. These impairments may increase the risk of operational errors underwater and represent an important physiological factor limiting air-diving depth and compromising diving safety (Rocco et al., 2019).

The mechanisms underlying nitrogen narcosis remain incompletely understood, and its assessment relies largely on subjective symptoms and behavioral manifestations that are susceptible to confounding factors and do not provide continuous, objective measures of brain function (van Wijk and Meintjes, 2014). Previous studies have suggested that these functional abnormalities may be associated with the effects of elevated PN_2_ on neuronal activity. At elevated partial pressures, nitrogen may interact with membrane lipids and proteins (Rostain and Lavoute, 2016), potentially modulating neurotransmitter receptors and associated ion channels, including γ-aminobutyric acid (GABA) and N-methyl-D-aspartate (NMDA) receptors (Rostain et al., 2011), with downstream effects on neuronal excitability and brain network activity. However, electrophysiological studies characterizing changes in brain function during hyperbaric nitrox exposure remain limited (Dean et al., 2003).

Electroencephalography (EEG) enables continuous recording of synchronized electrical activity generated by neuronal populations and provides an important neurophysiological measure of brain functional states (Purdon et al., 2015). Previous studies have shown that hyperbaric exposure to nitrogen-containing mixture may alter cerebral electrophysiological activity. Pastena et al. reported increased δ- and θ-wave activity in humans exposed to air at 900 kPa, accompanied by a shift in α-wave activity toward the frontal regions (Pastena et al., 2005). Vrijdag et al. demonstrated that hyperbaric air exposure at 608 kPa increased the global efficiency of EEG functional connectivity, whereas hyperbaric heliox exposure at the same pressure did not produce a statistically significant change relative to baseline (Vrijdag et al., 2022). These findings suggest that electrophysiological measures may reflect changes in brain function associated with hyperbaric exposure to nitrogen-containing mixture. However, most human studies have employed multichannel EEG monitoring before and after hyperbaric exposure or at selected time points, with analyses largely focusing on individual frequency-band power measures (Vrijdag et al., 2022). Consequently, the dynamic evolution and stage-dependent characteristics of electrophysiological changes during sustained exposure remain incompletely characterized.

Heliox is an important alternative breathing mixture to nitrox in deep diving because helium has a lower narcotic potency than nitrogen. Accordingly, this study was designed to delineate the stage-specific changes in cortical electrophysiological activity during hyperbaric nitrox exposure and subsequent hyperbaric heliox exposure. We hypothesized that hyperbaric nitrox exposure would be associated with a shift in ECoG activity toward lower frequencies and reduced signal complexity, whereas subsequent hyperbaric heliox exposure would attenuate these electrophysiological alterations and shift ECoG features toward baseline levels. By characterizing electrophysiological changes across different exposure stages, this study may provide an electrophysiological basis for further investigation on the relationship between hyperbaric nitrox exposure and nitrogen narcosis.

## 2 Materials and methods

### 2.1 Animals and grouping

A total of 24 male Sprague–Dawley rats (230–250 g; Shanghai BK Laboratory Animal Co., Ltd., Shanghai, China) were used in this study. Before the experiments, all animals were acclimated for 2 days under controlled conditions (22 ± 2 °C, 60%–70% relative humidity, and a 12 h light/dark cycle), with *ad libitum* access to food and water. Following ECoG electrode implantation, the animals were allowed to recover for 7 days and subsequently randomly assigned to the 16% O_2_–N_2_, 3% O_2_–N_2_, and 100% O_2_ group using a random-sequence generation method (n=8 per group). Before gas exposure, baseline assessments, including body weight, electrode implantation status, and pre-exposure ECoG recordings, were performed in all animals. All experimental procedures were approved by the Animal Ethics Committee of Naval Medical Center, Naval Medical University.

### 2.2 Electrode implantation

Electrode implantation was performed as previously described (Pearce et al., 2014). Under isoflurane anesthesia, rats were secured in a stereotaxic apparatus (SA-150; Shanghai Yuyan Scientific Instrument Co., Ltd., Shanghai, China), and ECoG electrodes were implanted under aseptic conditions. Using bregma as the stereotaxic reference, three electrodes were implanted over the right frontal cortex (AP + 2.0 mm, ML + 1.5 mm), left parietal cortex (AP −3.8 mm, ML −2.0 mm), and cerebellum (AP −7.0 mm, ML 0 mm; ground electrode), respectively. The right frontal and left parietal electrodes served as the positive and negative inputs for differential recording, respectively, while the cerebellar electrode served as the ground electrode, forming a single-channel ECoG recording configuration. This frontal–parietal differential configuration was selected to provide a stable cortical potential difference for continuous ECoG monitoring during hyperbaric exposure. Following electrode fixation, the animals were housed individually and allowed to recover for 7 days to minimize the potential effects of surgery and anesthesia on subsequent ECoG recordings.

### 2.3 Hyperbaric exposure

Seven days after electrode implantation, rats were placed in an animal hyperbaric chamber (Wuhu Diving Equipment Factory Co., Ltd., Shanghai Salvage Bureau, Shanghai, China). The electrodes were connected via lead wires to a multichannel physiological data acquisition system (PowerLab with LabChart 8; ADInstruments Pty Ltd., Sydney, Australia) for continuous ECoG recording. ECoG signals were acquired continuously at a sampling frequency of 400 Hz. Although the system supported multichannel acquisition, only a single differential ECoG channel was recorded in this study. Before pressurization, ECoG activity was continuously recorded under normobaric conditions, and a stable 10-min pre-exposure segment was selected as the baseline for analysis.

Rats in the 16% O_2_–N_2_ group were pressurized to 700 kPa with a mixture containing 16% O_2_ and 84% N_2_ (nitrox) at a rate of 100 kPa/min. Following 60 min of exposure at 700 kPa, the nitrox was replaced within 5 min with a mixture containing 84% He and 16% O_2_ (heliox) at the same pressure, and the animals were exposed for an additional 60 min. Rats in the 3% O_2_–N_2_ group were initially pressurized with air to 600 kPa at a rate of 100 kPa/min over approximately 5 min, and then the hyperbaric air in the chamber was replaced with a mixture containing 3% O_2_ and 97% N_2_. Once the concentration of oxygen in the chamber decreased to approximately 3%, rats were further pressurized to 700 kPa which was maintained for 60 min. Rats in the 100% O_2_ group were pressurized to 200 kPa with pure oxygen, which was maintained for 60 min.

Throughout hyperbaric exposure, the chamber was continuously ventilated to maintain the target oxygen concentration. ECoG activity was recorded continuously, while animal behaviors were simultaneously observed and documented. Behavioral observations were used solely for general assessment of animals’ condition during the experiment. As no predefined quantitative behavioral scoring system was established, these observations were not included in the statistical analysis. At the end of exposure, the chamber was depressurized to 100 kPa at a rate of 100 kPa/min.

### 2.4 Preprocessing and exposure-phase segmentation of ECoG

The ECoG signals were band-pass filtered at 0.5–100 Hz using a fourth-order Butterworth filter, and 50-Hz power-line interference was attenuated using an infinite impulse response (IIR) notch filter with a quality factor of Q = 30. Both filters were applied bidirectionally to achieve zero-phase filtering. Artifact-contaminated intervals were manually identified based on obvious movement-related interference, abrupt large-amplitude signal disturbances, or marked baseline drift inconsistent with the surrounding ECoG activity. These intervals were excluded from subsequent analyses before epoch segmentation. The remaining contiguous artifact-free segments were then divided separately into non-overlapping 10-s epochs for ECoG feature extraction.

The 60-min exposure period was divided into six consecutive 10-min intervals: 0–10 min, 10–20 min, 20–30 min, 30–40 min, 40–50 min, and 50–60 min. The stable pre-exposure recording period, as defined by the experimental time markers, was used as the baseline. All ECoG metrics were calculated for each 10-s epoch and subsequently averaged within each experimental phase for each animal. The resulting animal-level phase means were used as the observations for statistical analysis.

### 2.5 Power spectral and frequency-band power analysis

Power spectral density (PSD) was estimated for each valid 10-s epoch using Welch’s method with a Hann window, a subwindow length of 4 s, and 50% overlap between adjacent subwindows. The frequency range analyzed was 1–100 Hz. Frequency bands were defined as δ (1–4 Hz), θ (4–8 Hz), α (8–12 Hz), β (12–30 Hz), and γ (30–80 Hz). Absolute power in each frequency band was calculated by integrating PSD over the corresponding frequency range. For the γ band, the 48–52 Hz range was excluded to minimize the influence of local spectral attenuation caused by the 50 Hz notch filter on power estimation. Absolute frequency-band power was averaged across all valid 10-s epochs within each experimental phase for each animal. The change in power relative to baseline was then calculated as follows:


$$\Delta {\text{P}}_{\text{dB}}=\frac{10{\text{log}}_{10}({\text{P}}_{\text{phase}})}{{\text{P}}_{\text{baseline}}}$$

Here, *P*_phase_ and *P*_baseline_ represent the mean absolute power in the same frequency band for the same animal during the corresponding experimental phase and baseline period, respectively. Positive values indicate power above baseline, whereas negative values indicate power below baseline.

### 2.6 Principal ECoG features

The delta–theta/alpha–beta power ratio (DTABR), median frequency (MF), sample entropy (SampEn), and Higuchi fractal dimension (HFD) were selected as the principal ECoG features. DTABR and MF characterize the spectral distribution of ECoG activity, whereas SampEn and HFD quantify temporal regularity and scale-dependent geometric irregularity, respectively.

(1) DTABR

To reduce the skewness of ratio distribution, the DTABR was log-transformed using base 10 as follows:


$$R_{\log}=\log_{10}(\frac{P_{\delta }+P_{\theta }}{P_{\alpha }+P_{\beta }})$$

Here, *P*_δ_, *P*_θ_, *P*_α_, and *P*_β_ represent the absolute power in the corresponding frequency bands, respectively. An increase in *R*_log_ indicates a relative increase in δ- and θ-band power compared with α- and β-band power; *R*_log_ = 0 indicates equal summed power, whereas positive values indicate predominance of δ- and θ-band power and negative values indicate predominance of α- and β-band power.

(2) MF

MF was defined as the frequency below which 50% of the total spectral power within the 1–100 Hz range was contained. A decrease in MF indicates an overall shift of the spectral power distribution toward lower frequencies, whereas an increase indicates a shift toward higher frequencies.

(3) SampEn

SampEn was calculated for each valid 10-s epoch after resampling the signal from 400 Hz to 200 Hz. The embedding dimension *m* was set to 2, and the similarity tolerance *r* was set to 0.2 times the standard deviation (SD) of corresponding epoch, i.e., *r* = 0.2 × SD (Richman and Moorman, 2000). SampEn was defined as the negative natural logarithm of the conditional probability that patterns similar for *m* consecutive samples remain similar when extended by one additional sample, with self-matches excluded from the calculation. A decrease in SampEn indicates increased repetition of local temporal patterns, greater signal regularity, and enhanced short-term predictability, whereas an increase indicates reduced pattern repetition, greater signal complexity and irregularity, and lower short-term predictability.

(4) HFD

HFD was calculated from signals resampled to 200 Hz, with the maximum scale *K*_max_ set to 10 (Schmidig et al., 2024). HFD was estimated by calculating the normalized curve length *L(k)* at different sampling intervals *k* and determining the linear relationship between ln[*L(k)*] and ln(1/*k*). A decrease in HFD indicates reduced multiscale geometric irregularity and an overall smoother waveform, whereas an increase indicates greater multiscale geometric irregularity and a rougher waveform.

### 2.7 Animal-level data aggregation

For each valid 10-s epoch, the power spectrum, absolute frequency-band power, and four principal ECoG features, including log-transformed DTABR, MF, SampEn, and HFD, were calculated. The values obtained from all valid epochs within each experimental phase were then averaged for each animal to derive an animal-level value for that phase. Changes in absolute frequency-band power relative to baseline were expressed using the dB transformation described above, whereas baseline-relative changes in the remaining ECoG features were calculated as follows:


$$\Delta X_{i,j}=X_{i,j}-X_{i,baseline}$$

Here, $X_{i,j}$ denotes the value of the corresponding metric for animal *i* during experimental phase *j*, and $X_{i,baseline}$ denotes the baseline value for the same animal. Each animal was treated as an independent experimental unit for statistical analysis, whereas individual 10-s epochs were used only to calculate phase-level means and were not treated as independent statistical observations.

### 2.8 Statistical analysis

Each animal was treated as an independent experimental unit, with eight animals per group. Individual 10-s epochs were not considered independent statistical observations. Continuous variables are presented as the median (first quartile, third quartile) [M (Q1, Q3)]. Given the relatively small sample size and the potentially different distributional characteristics of the ECoG measures, two-sided nonparametric methods were prespecified for statistical inference.

Overall intragroup differences across experimental phases were assessed using the Friedman test, with Kendall’s W reported as the effect size. Comparisons between individual phases and baseline or other paired conditions were performed using the Wilcoxon signed-rank test. Overall differences among the three groups were assessed using the Kruskal–Wallis test, followed by pairwise Mann–Whitney U tests when the overall test reached statistical significance. Categorical variables were analyzed using the Fisher–Freeman–Halton exact test, with pairwise Fisher’s exact tests performed when appropriate. For paired comparisons assessed using the Wilcoxon signed-rank test, the rank-biserial correlation coefficient (*r_rb_*) was calculated directly from the signed ranks as an effect-size measure. The sign of *r_rb_* indicates the direction of the paired difference, whereas |*r_rb_*| reflects the magnitude of the effect, with values closer to 1 indicating a stronger effect across paired animal-level observations. For an exploratory *post-hoc* analysis, the 30–40 min phase of hyperbaric nitrox exposure in the 16% O_2_-N_2_ group was selected as the reference phase based on the observed phase-specific patterns in DTABR, MF, SampEn, and HFD. Subsequent hyperbaric heliox exposure phases were then compared with it.

P values from multiple comparisons were adjusted using the Benjamini–Hochberg (BH) procedure. Adjusted values were denoted as PBH, with PBH < 0.05 considered statistically significant. For intragroup stage-specific comparisons, the six comparisons between each exposure stage and baseline for each metric within each experimental group were treated as a separate family of hypotheses and subjected to BH correction. For the overall time effect of frequency-band power, the 15 Friedman tests corresponding to 3 experimental groups × 5 frequency bands were treated as a separate hypothesis family and subjected to BH correction. Details of data normalization, specific comparison schemes, and the scope of BH correction for each analysis are provided in the **Supplementary Materials**. All statistical analyses were performed using Python (version 3.9.18; Python Software Foundation, USA).

## 3 Results

### 3.1 Changes in ECoG during hyperbaric nitrox exposure

#### 3.1.1 Changes in ECoG spectral structure

Valid ECoG data were obtained from all rats during the pre-exposure baseline period and all six exposure phases in each group. Compared with baseline, rats in both the 3% O_2_–N_2_ and 16% O_2_–N_2_ groups exhibited increased ECoG amplitudes and enhanced slow-wave activity at 700 kPa, whereas the overall waveform morphology remained largely unchanged in the 100% O_2_ group (**Figure 1A**). Time–frequency analysis revealed an early increase in low-frequency power at 10–20 min in the 3% O_2_–N_2_ group, followed by phase-dependent fluctuations. In the 16% O_2_–N_2_ group, the increase in low-frequency power became apparent primarily during the middle-to-late exposure period (approximately 40 min), whereas the time–frequency pattern remained generally stable in the 100% O_2_ group (**Figure 1B**). Further examination of the PSD curves showed that power at lower frequencies exceeded baseline levels in both the 3% O_2_–N_2_ and 16% O_2_–N_2_ groups, whereas power at frequencies above approximately 20–30 Hz progressively declined below baseline levels during exposure. In contrast, the PSD profile in the 100% O_2_ group remained generally similar to that at baseline (**Figure 1C**).

**Figure 1:**
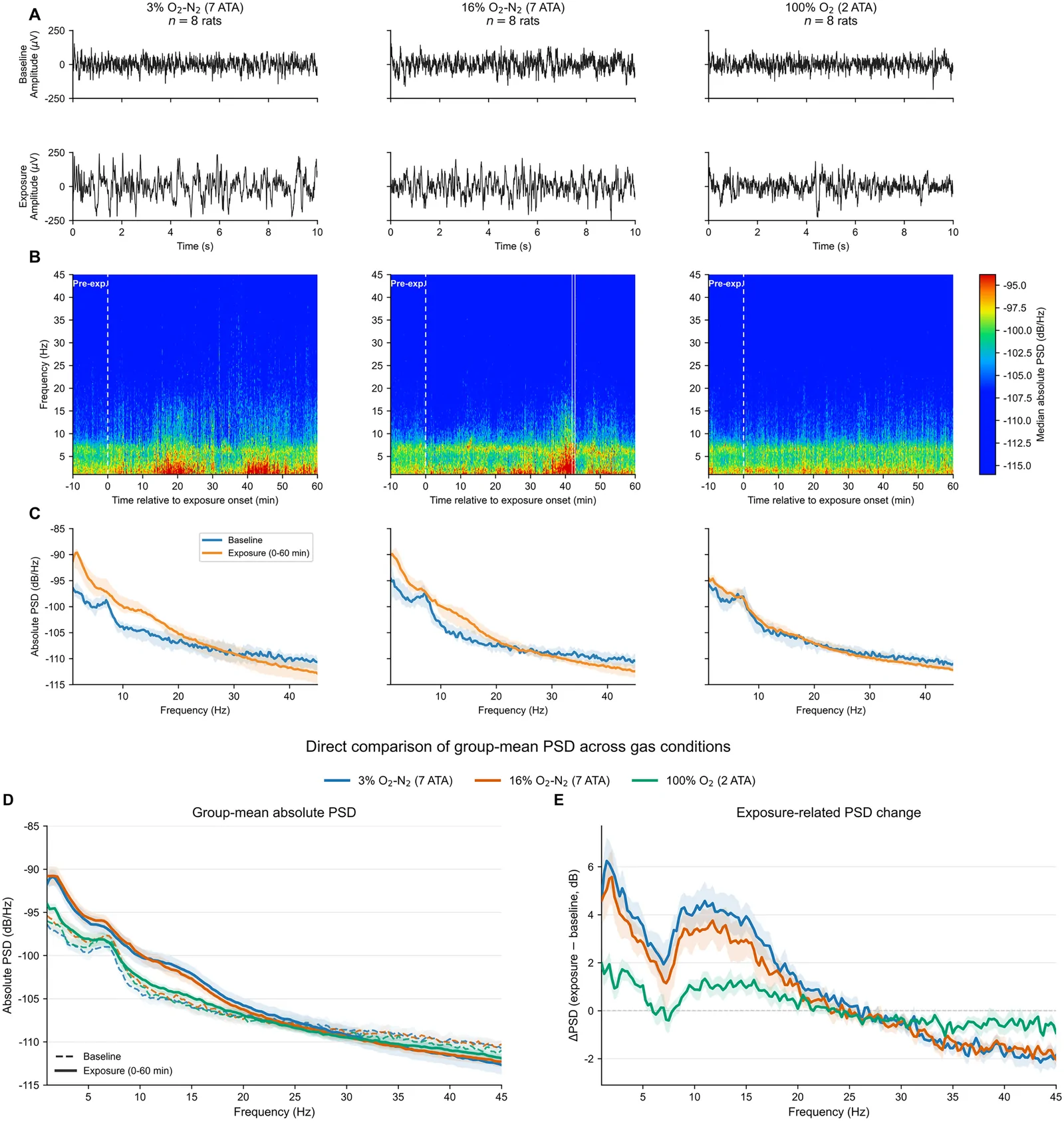
ECoG waveforms, time–frequency distributions, and power spectral density profiles in the three groups. **(A)** Representative 10-s ECoG waveforms at pre-exposure baseline and exposure periods in each group. **(B)** Time–frequency maps of absolute power spectral density from 10 min before exposure to 60 min after exposure onset; color represents the group median across animals, and the white dashed line indicates the onset of exposure. **(C)** Power spectral density during baseline and the 0–60 min exposure period; solid lines represent the median and shaded areas represent Q1–Q3. **(D)** Overall mean absolute power spectral density during baseline and exposure; dashed and solid lines represent baseline and exposure, respectively, and shaded areas represent Q1–Q3. **(E)** Baseline-relative changes in power spectral density during exposure; curves represent the median paired difference and shaded areas represent Q1–Q3, with positive and negative values indicating power above and below baseline, respectively. n = 8 per group; only the 1–45 Hz frequency range is shown in **(B–E)**.

Baseline-normalized PSD analysis showed increased low-frequency power in both 3% O_2_–N_2_ and 16% O_2_–N_2_ groups, whereas the PSD profile in the 100% O_2_ group remained closer to baseline levels. With increasing frequency, the curves for all three groups gradually approached baseline levels, reaching values close to zero at approximately 23–27 Hz before becoming negative. At higher frequencies, both 3% O_2_–N_2_ and 16% O_2_–N_2_ groups exhibited varying degrees of power reduction, whereas the 100% O_2_ group remained relatively stable overall (**Figures 1D, E**).

#### 3.1.2 Absolute frequency-band power

In the 3% O_2_–N_2_ group, δ-, θ-, α-, and β-band power generally increased, whereas γ-band power decreased. During exposure, the median baseline-relative changes in δ-, θ-, α-, β-, and γ-band power ranged from 2.95 to 6.06 dB, 1.92 to 3.87 dB, 2.32 to 4.70 dB, 1.10 to 2.45 dB, and −2.94 to −1.05 dB, respectively. Overall analysis showed the power of all five frequency bands changed significantly over time in the 3% O_2_–N_2_ group (δ: χ²(6) = 22.82, *P*_BH_ = 0.0064, W = 0.48; θ: χ²(6) = 15.27, *P*_BH_ = 0.0372, W = 0.32; α: χ²(6) = 19.45, *P*_BH_ = 0.0130, W = 0.41; β: χ²(6) = 18.75, *P*_BH_ = 0.0138, W = 0.39; γ: χ²(6) = 21.11, *P*_BH_ = 0.0088, W = 0.44). Phase-specific analysis showed that, compared with baseline, δ-band power was significantly increased at 0–10 min and 50–60 min (both *P*_BH_ = 0.0469), 10–20 min and 20–30 min (both *P*_BH_ = 0.0234), and 40–50 min (*P*_BH_ = 0.0313); θ- and α-band power was significantly increased at 10–20 min, 20–30 min, and 40–50 min (all *P*_BH_ = 0.0469); β-band power was significantly increased at 10–20 min and 40–50 min (both *P*_BH_ = 0.0234); γ-band power was significantly decreased at 0–10 min, 10–20 min, and 20–30 min (all *P*_BH_ = 0.0156), 30–40 min (*P*_BH_ = 0.0469), and 40–50 min (*P*_BH_ = 0.0234) (**Figure 2A**).

**Figure 2:**
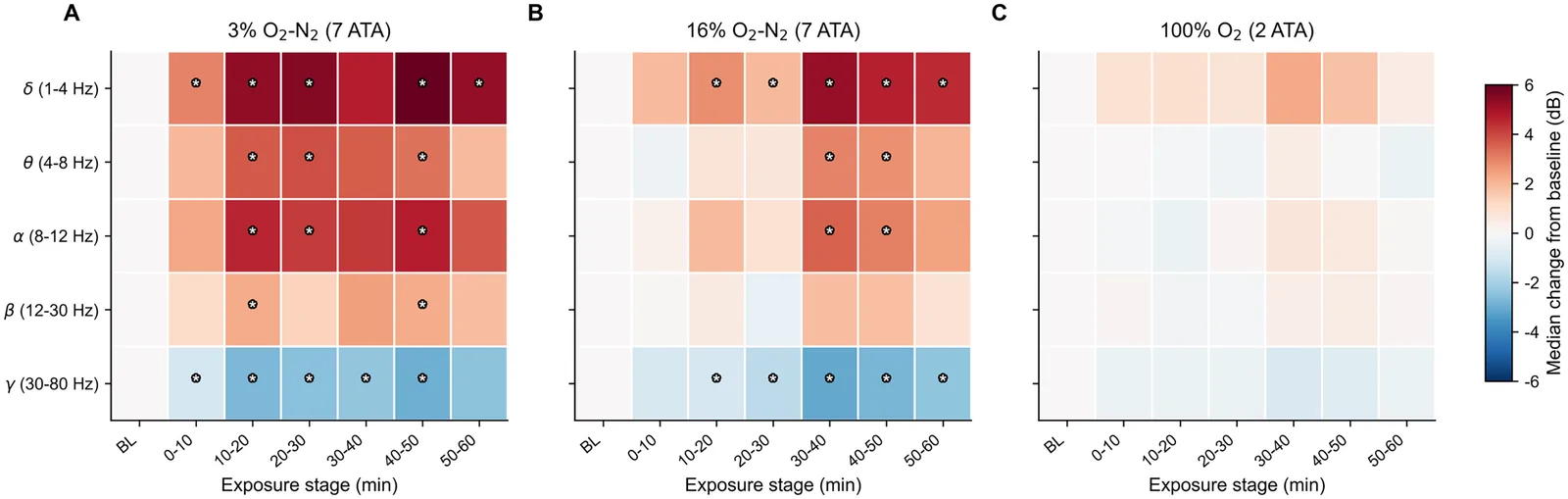
Baseline-normalized changes in absolute frequency-band power during hyperbaric nitrox exposure. **(A)** 3% O_2_–N_2_ group at 7 ATA; **(B)** 16% O_2_–N_2_ group at 7 ATA; **(C)** 100% O_2_ group at 2 ATA. Colors represent the median baseline-relative changes in absolute frequency-band power at each exposure phase, expressed in dB. Red indicates increased power, whereas blue indicates decreased power. BL, baseline; *P_BH_ < 0.05; n = 8 per group.

In the 16% O_2_–N_2_ group, δ-, θ-, α-, and β-band power generally increased, whereas γ-band power decreased. Across the six exposure phases, the median baseline-relative changes in δ-, θ-, α-, β-, and γ-band power ranged from 1.88 to 5.22 dB, −0.32 to 2.99 dB, 0.28 to 3.55 dB, −0.43 to 1.84 dB, and −3.07 to −0.94 dB, respectively. Overall analysis showed the δ-, θ-, and γ-band power changes significantly over time in the 16% O_2_–N_2_ group (δ: χ²(6) = 16.39, *P*_BH_ = 0.0295, W = 0.34; θ: χ²(6) = 15.05, *P*_BH_ = 0.0372, W = 0.31; γ: χ²(6) = 26.04, *P*_BH_ = 0.0033, W = 0.54), whereas α-band power (χ²(6) = 10.07, *P*_BH_ = 0.1825, W = 0.21) and β-band power (χ²(6) = 5.84, *P*_BH_ = 0.4730, W = 0.12) remained stable over time. Phase-specific analysis showed that, compared with baseline, δ-band power was significantly increased at 10–20 min (*P*_BH_ = 0.0234), 20–30 min, 30–40 min, and 40–50 min (all *P*_BH_ = 0.0156), and 50–60 min (*P*_BH_ = 0.0469). Both θ- and α-band power was significantly increased at 30–40 min and 40–50 min (all *P*_BH_ = 0.0234). γ-band power was significantly decreased at 10–20 min (*P*_BH_ = 0.0469), 20–30 min and 30–40 min (both *P*_BH_ = 0.0156), 40–50 min (*P*_BH_ = 0.0234), and 50–60 min (*P*_BH_ = 0.0156). β-band power remained stable at baseline and six exposure phases (all *P*_BH_ > 0.05) (**Figure 2B**).

In the 100% O_2_ group, changes in power across all frequency bands were relatively small. Across six exposure phases, the median relative changes in δ-, θ-, α-, β-, and γ-band power ranged from 0.51 to 2.28 dB, −0.39 to 0.44 dB, −0.34 to 0.73 dB, −0.21 to 0.45 dB, and −0.94 to −0.35 dB, respectively. Overall analysis showed the power of any frequency band remained stable over time in the 100% O_2_ group (δ: χ²(6) = 10.29, *P*_BH_ = 0.1825, W = 0.21; θ: χ²(6) = 3.75, *P*_BH_ = 0.7105, W = 0.08; α: χ²(6) = 9.70, *P*_BH_ = 0.1882, W = 0.20; β: χ²(6) = 8.46, *P*_BH_ = 0.2575, W = 0.18; γ: χ²(6) = 8.14, *P*_BH_ = 0.2629, W = 0.17). Phase-specific analysis also showed no significant differences in any frequency-band power at baseline and any exposure phase (**Figure 2C**).

The mean changes of five frequency bands across six exposure phases relative to the baseline levels were calculated for each rat, and then M (Q1, Q3) was determined for each group according to the value of each rat.

Comparisons of three groups revealed significant differences in the δ-, θ-, α-, β-, and γ-band power (*P*_BH_ = 0.0213, 0.0210, 0.0086, 0.0427, and 0.0213, respectively). Compared with 100% O_2_ group, the δ-, θ-, α-, and β-band power was significantly increased (*P*_BH_ = 0.0156, 0.0312, 0.0019 and 0.0312, respectively) and the γ-band power was significantly decreased (*P*_BH_ = 0.0210) in the 3% O_2_–N_2_ group. The 16% O_2_–N_2_ group also exhibited significantly higher δ-, θ-, and α-band power (*P*_BH_ = 0.0156, 0.0422 and 0.0044, respectively) and markedly lower γ-band power (*P*_BH_ = 0.0310) as compared to 100% O_2_ group. No significant difference was observed in the β-band power between 16% O_2_–N_2_ group and 100% O_2_ group (*P*_BH_ = 0.2786). No significant differences were found in any frequency-band power between 3% O_2_–N_2_ group and 16% O_2_–N_2_ group (all *P*_BH_ > 0.05) (**Figure 3A**).

**Figure 3:**
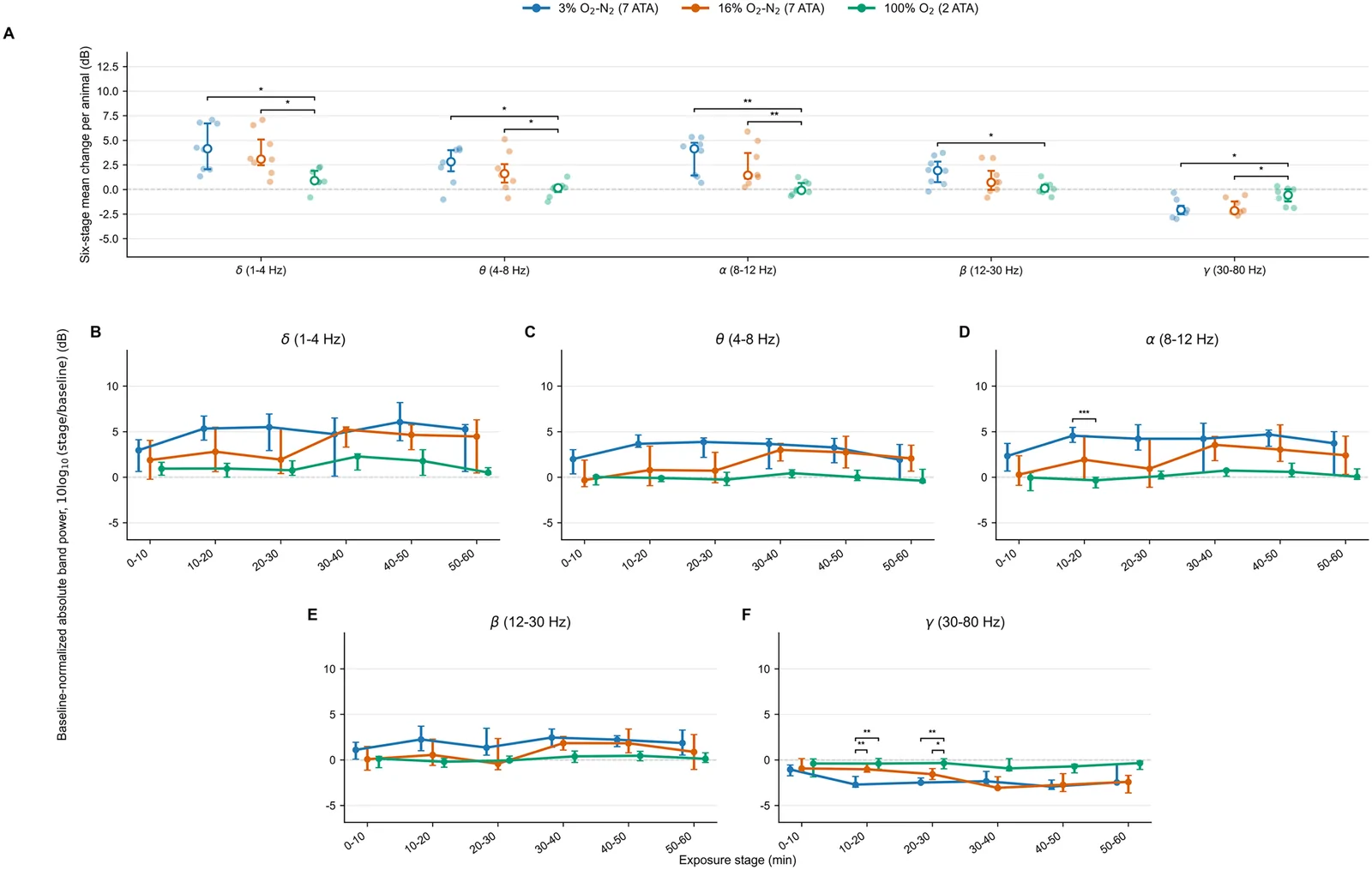
Overall and phase-specific intergroup comparisons of baseline-normalized absolute ECoG frequency-band power. Data for each rat were normalized to its own baseline and expressed in dB. **(A)** Overall comparison among three groups; light-colored dots represent individual rats, whereas open circles and error bars represent the median and Q1–Q3, respectively. **(B–F)** Intergroup comparisons of δ-, θ-, α-, β-, and γ-band power, respectively, across exposure phases; colored lines and circles represent the median, and error bars represent Q1–Q3. *P_BH_ < 0.05, **P_BH_ < 0.01, and *** P_BH_ < 0.001; n = 8 per group.

At 10–20 min, α-band power differed significantly between 3% O_2_–N_2_ group and 100% O_2_ group (*P*_BH_ = 0.0448), with median changes [M (Q1, Q3)] of 4.55 (3.81, 5.47) dB and −0.34 (−1.18, −0.01) dB, respectively. At both 10–20 and 20–30 min, γ-band power differed significantly between 3% O_2_–N_2_ group and 100% O_2_ group (both *P*_BH_ = 0.0448). At 10–20 min, the median changes in γ-band power were −2.72 (−2.99, −1.82) dB in the 3% O_2_–N_2_ group and −0.41 (−1.03, 0.16) dB in the 100% O_2_ group; at 20–30 min, the median changes in γ-band power were −2.50 (−2.61, −1.98) dB and −0.35 (−0.99, 0.13) dB, respectively. No significant inter-group differences were observed for the remaining frequency bands or exposure phases (all *P*_BH_ > 0.05) (**Figures 3B–F**).

#### 3.1.3 Temporal changes in principal ECoG features

In both 3% O_2_–N_2_ group and 16% O_2_–N_2_ group, DTABR generally increased, whereas MF, SampEn, and HFD generally decreased; comparatively smaller changes were observed in the 100% O_2_ group. In both 3% O_2_–N_2_ and 16% O_2_–N_2_ groups, DTABR generally increased, whereas MF, SampEn, and HFD generally decreased (**Figures 4A–D**).

**Figure 4:**
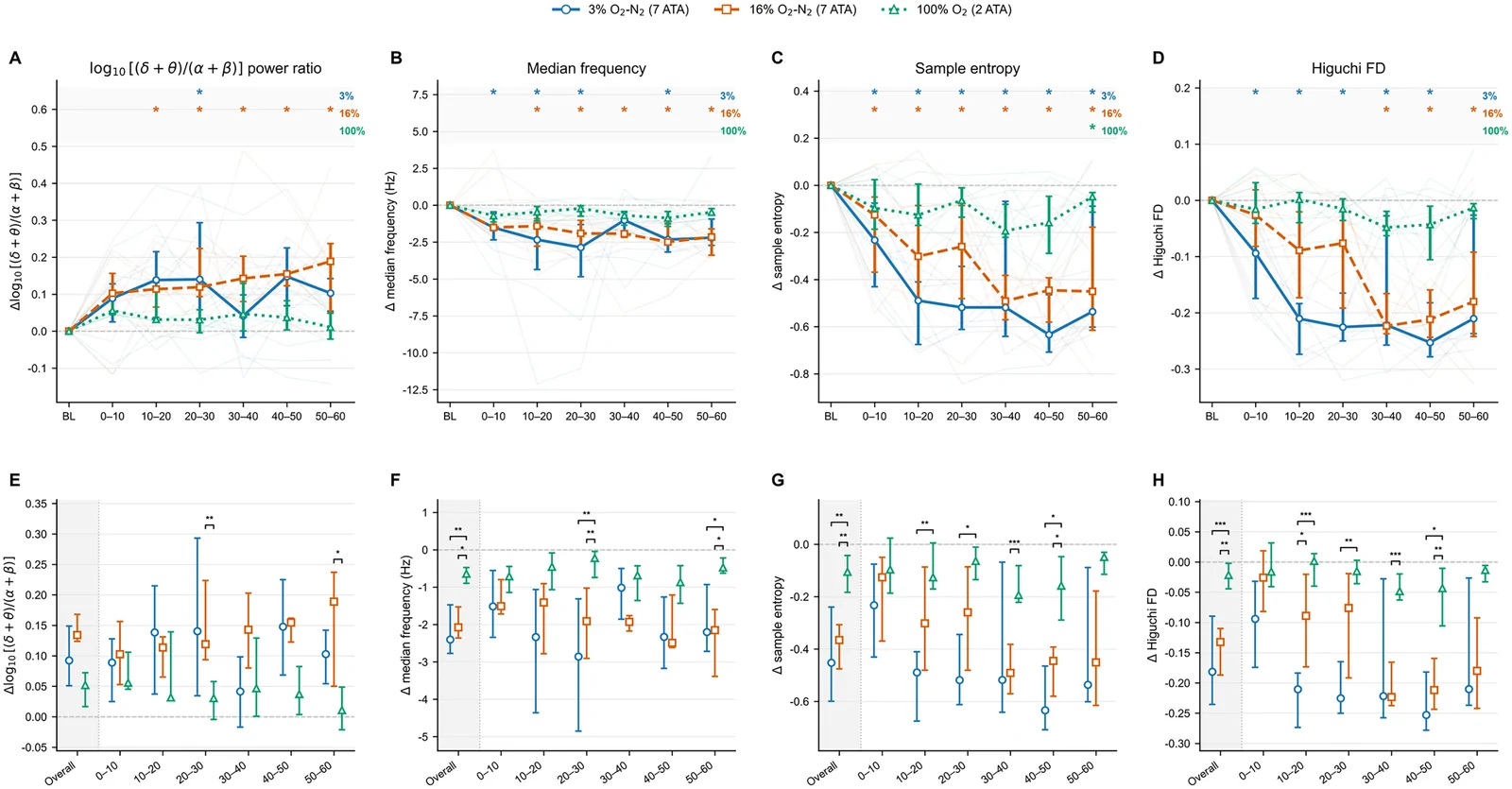
Changes in principal ECoG features across exposure phases in three groups. **(A–D)** Temporal changes in DTABR, MF, SampEn, and HFD during exposure. Thin gray lines represent individual rats, and colored thick lines represent the median and interquartile range (Q1–Q3); P_BH_ < 0.05 *vs*. baseline. **(E–H)** Overall and phase-specific intergroup comparisons of DTABR, MF, SampEn, and HFD, respectively. “Overall” represents the mean baseline-relative change across the six exposure phases for each rat, whereas the remaining columns represent the baseline-relative change during the corresponding exposure phase. Colored symbols and error bars represent the median and interquartile range (Q1–Q3), respectively. *P_BH_ < 0.05, **P_BH_ < 0.01, *** P_BH_ < 0.001. BL, baseline; n = 8.

In the 3% O_2_–N_2_ group, MF (χ²(6) = 24.11, *P*_BH_ = 0.0045, Kendall’s W = 0.50), SampEn (χ²(6) = 25.07, *P*_BH_ = 0.0045, Kendall’s W = 0.52) and HFD (χ²(6) = 20.73, *P*_BH_ = 0.0072, Kendall’s W = 0.43) changed significantly over time during the exposure. DTABR showed an increasing trend, but the change over time did not reach statistical significance (χ²(6) = 13.13, *P*_BH_ = 0.0616, Kendall’s W = 0.27). Phase-specific comparisons showed that DTABR only at 20–30 min was significantly higher than baseline level (*P*_BH_ = 0.0469). MF at 0–10, 10–20, 20–30, and 40–50 min was significantly lower than baseline level (*P*_BH_ = 0.0156, 0.0156, 0.0156, and 0.0234, respectively). SampEn at all exposure phases was significantly lower than baseline level (*P*_BH_ = 0.0188, 0.0117, 0.0117, 0.0117, 0.0117, and 0.0391, respectively). HFD was significantly lower than baseline at 0–10 min, 10–20 min, 20–30 min, 30–40 min and 40–50 min (*P*_BH_ = 0.0352, 0.0234, 0.0234, 0.0469, and 0.0313, respectively).

In the 16% O_2_–N_2_ group, DTABR (χ²(6) = 17.14, *P*_BH_ = 0.0167, Kendall’s W = 0.36), MF (χ²(6) = 15.27, *P*_BH_ = 0.0295, Kendall’s W = 0.32), SampEn (χ²(6) = 17.46, *P*_BH_ = 0.0162, Kendall’s W = 0.36) and HFD (χ²(6) = 18.21, *P*_BH_ = 0.0150, Kendall’s W = 0.38) changed significantly over time during the exposure. DTABR was significantly higher than baseline level from 10–20 min onward and remained elevated through 50–60 min (*P*_BH_ = 0.0281, 0.0156, 0.0156, 0.0156, and 0.0234, respectively). MF was significantly lower than baseline level from 10–20 min through 50–60 min (*P*_BH_ = 0.0281, 0.0156, 0.0156, 0.0156, and 0.0234, respectively). SampEn was significantly lower than baseline level at all exposure phases (*P*_BH_ = 0.0391, 0.0391, 0.0156, 0.0156, 0.0156, and 0.0234, respectively). HFD was significantly lower than baseline level at 30–40 min, 40–50 min and 50–60 min (*P*_BH_ = 0.0234, 0.0234, and 0.0469, respectively).

In the 100% O_2_ group, DTABR changed significantly over time (χ²(6) = 18.70, *P*_BH_ = 0.0141, Kendall’s W = 0.39), although it at exposure phases did not differ significantly from baseline level (all *P*_BH_ > 0.05). MF (χ²(6) = 9.97, *P*_BH_ = 0.1651, Kendall’s W = 0.21), SampEn (χ²(6) = 8.30, *P*_BH_ = 0.2677, Kendall’s W = 0.17), and HFD (χ²(6) = 6.96, *P*_BH_ = 0.3583, Kendall’s W = 0.15) showed no significant changes over time. Although SampEn showed no significant changes over time, it at 50–60 min was significantly lower than baseline level (*P*_BH_ = 0.0469).

In addition, the mean change across six exposure phases was calculated for each rat as compared to the baseline level. Significant differences were observed for MF (*P*_BH_ = 0.0081), SampEn (*P*_BH_ = 0.0081), and HFD (*P*_BH_ = 0.0054) between groups, whereas no significant difference was found for DTABR (*P*_BH_ = 0.0604) between groups. Pairwise comparisons showed that MF was significantly lower in both 3% O_2_–N_2_ group and 16% O_2_–N_2_ group than in 100% O_2_ group (*P*_BH_ = 0.0089 and 0.0156, respectively). SampEn was significantly lower in both 3% O_2_–N_2_ group and 16% O_2_–N_2_ group than in 100% O_2_ group (both *P*_BH_ = 0.0070). HFD was also significantly lower in both 3% O_2_–N_2_ group and 16% O_2_–N_2_ group than in 100% O_2_ group (*P*_BH_ = 0.0009 and 0.0044, respectively). No significant differences in MF, SampEn, or HFD were observed between 3% O_2_–N_2_ group and 16% O_2_–N_2_ group (all *P*_BH_ > 0.05) (**Figures 4E–H**).

Phase-specific comparisons revealed significant inter-group differences in DTABR at 20–30 min and 50–60 min (*P*_BH_ = 0.0404 and 0.0449, respectively), with DTABR in the 16% O_2_–N_2_ group being significantly higher than in the 100% O_2_ group (*P*_BH_ = 0.0056 and 0.0443, respectively). Significant inter-group difference in MF was observed at 20–30 min and 50–60 min (both *P*_BH_ = 0.0387), and MF was significantly lower in both 3% O_2_–N_2_ group and 16% O_2_–N_2_ group than in 100% O_2_ group at 20–30 min (both *P*_BH_ = 0.0070) and 50–60 min (both *P*_BH_ = 0.0156). Significant inter-group difference in SampEn was found at 10–20 min, 20–30 min, 30–40 min and 40–50 min (all *P*_BH_ = 0.0387); SampEn was significantly lower in the 3% O_2_–N_2_ group than in the 100% O_2_ group at 10–20 min and 20–30 min (*P*_BH_ = 0.0019 and 0.0140, respectively); it was lower in the 16% O_2_–N_2_ group than in the 100% O_2_ group at 30–40 min (*P*_BH_ = 0.0005); it was lower in both 3% O_2_–N_2_ group and 16% O_2_–N_2_ group than in 100% O_2_ group at 40–50 min (*P*_BH_ = 0.0310 and 0.0210, respectively). Significant inter-group difference in HFD was observed at 10–20 min, 20–30 min, 30–40 min, and 40–50 min (all *P*_BH_ = 0.0387); at 10–20 min, HFD was significantly lower in the 3% O_2_–N_2_ group than in both 16% O_2_–N_2_ group and 100% O_2_ group (*P*_BH_ = 0.0422 and 0.0009, respectively); at 20–30 min, it was significantly lower in the 3% O_2_–N_2_ group than in the 100% O_2_ group (*P*_BH_ = 0.0089); at 30–40 min, it was significantly lower in the 16% O_2_–N_2_ group than in the 100% O_2_ group (*P*_BH_ = 0.0005); and at 40–50 min, it was significantly lower in both 3% O_2_–N_2_ group and 16% O_2_–N_2_ group than in 100% O_2_ group (*P*_BH_ = 0.0221 and 0.0056, respectively) (**Figures 4E–H**).

#### 3.1.4 Consistency of direction of ECoG changes

In the 3% O_2_–N_2_ group, phase-specific *r_rb_* values ranged from 0.50 to 1.00 for DTABR, −1.00 to −0.56 for MF, −1.00 to −0.83 for SampEn, and −1.00 to −0.78 for HFD. Across six exposure phases, DTABR increased in 6/8, 6/8, 8/8, 6/8, 7/8 and 7/8 rats, respectively; MF decreased in 8/8, 8/8, 8/8, 6/8, 7/8 and 7/8 rats, respectively; SampEn decreased in 7/8, 8/8, 8/8, 8/8, 8/8, and 7/8 rats, respectively and HFD decreased in 7/8, 8/8, 8/8, 6/8, 7/8, and 6/8 rats, respectively (**Figure 5A**).

**Figure 5:**
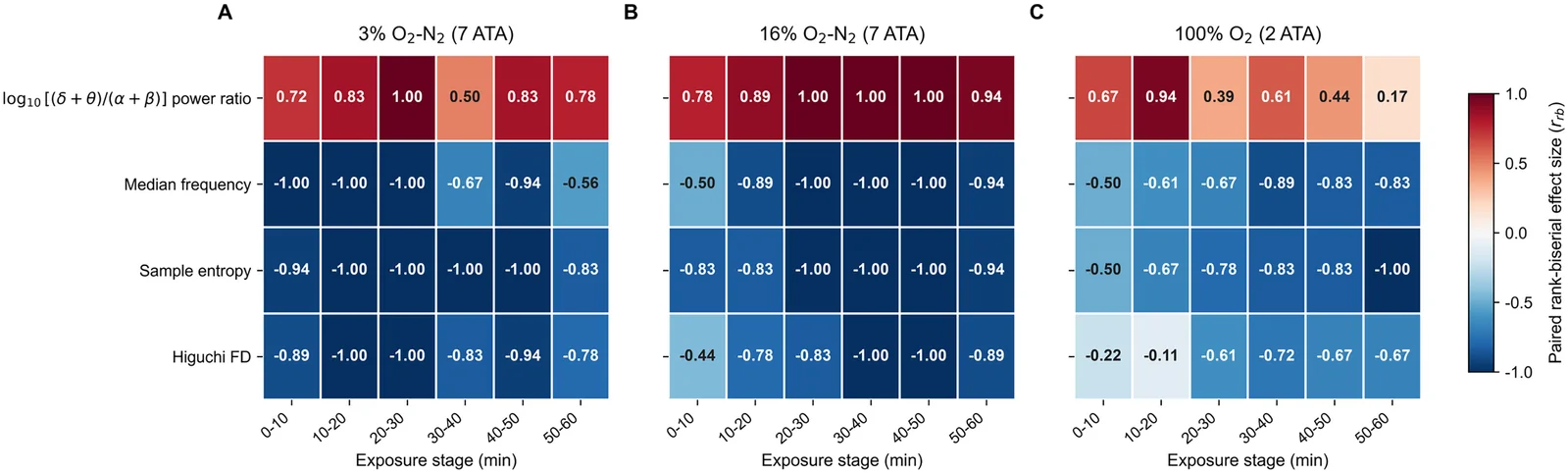
Direction of changes in principal ECoG features across exposure phases in different groups. **(A)** 3% O_2_–N_2_ group at 7 ATA; **(B)** 16% O_2_–N_2_ group at 7 ATA; **(C)** 100% O_2_ group at 2 ATA. Heat maps show *r*_rb_ values at each exposure phase relative to baseline. Red indicates an increase and blue indicates a decrease; positive values indicate that the feature increased in most rats, whereas negative values indicate that it decreased in most rats. The numbers within the cells are *r*_rb_ values; n = 8 per group.

In the 16% O_2_–N_2_ group, phase-specific *r_rb_* values ranged from 0.78 to 1.00 for DTABR, −1.00 to −0.50 for MF, −1.00 to −0.83 for SampEn, and −1.00 to −0.44 for HFD. Across six exposure phases, DTABR increased in 7/8, 7/8, 8/8, 8/8, 8/8, and 7/8 rats, respectively; MF decreased in 6/8, 7/8, 8/8, 8/8, 8/8, and 7/8 rats, respectively; SampEn decreased in 6/8, 7/8, 8/8, 8/8, 8/8, and 7/8 rats, respectively and HFD decreased in 5/8, 6/8, 6/8, 8/8, 8/8, and 7/8 rats, respectively (**Figure 5B**).

In the 100% O_2_ group, phase-specific *r_rb_* values ranged from 0.17 to 0.94 for DTABR, −0.89 to −0.50 for MF, −1.00 to −0.50 for SampEn, and −0.72 to −0.11 for HFD. Across six exposure phases, DTABR increased in 7/8, 7/8, 6/8, 6/8, 6/8, and 5/8 rats, respectively, MF decreased in 6/8, 6/8, 6/8, 7/8, 6/8, and 6/8 rats, respectively, SampEn decreased in 5/8, 6/8, 6/8, 6/8, 6/8, and 8/8 rats, respectively and HFD decreased in 5/8, 3/8, 6/8, 6/8, 6/8, and 7/8 rats, respectively (**Figure 5C**).

In the overall comparison, DTABR increased, and MF, SampEn and HFD decreased in 8/8 rats in both 3% O_2_–N_2_ group and 16% O_2_–N_2_ group, with corresponding *r_rb_* values of 1.00, −1.00, −1.00, and −1.00, respectively. In the 100% O_2_ group, DTABR increased in 6/8 rats, whereas MF, SampEn, and HFD decreased in 7/8 rats, with corresponding *r_rb_* values of 0.56, −0.94, −0.89, and −0.83, respectively. No significant differences in the overall direction of change were observed among three groups (all *P*_BH_ > 0.05) (**Figure 6A**). Phase-specific *r_rb_* values for DTABR, MF, SampEn, and HFD are shown in **Figures 6B–E**, and no significant differences in the direction of change were detected among three groups at any exposure phase (all *P*_BH_ > 0.05).

**Figure 6:**
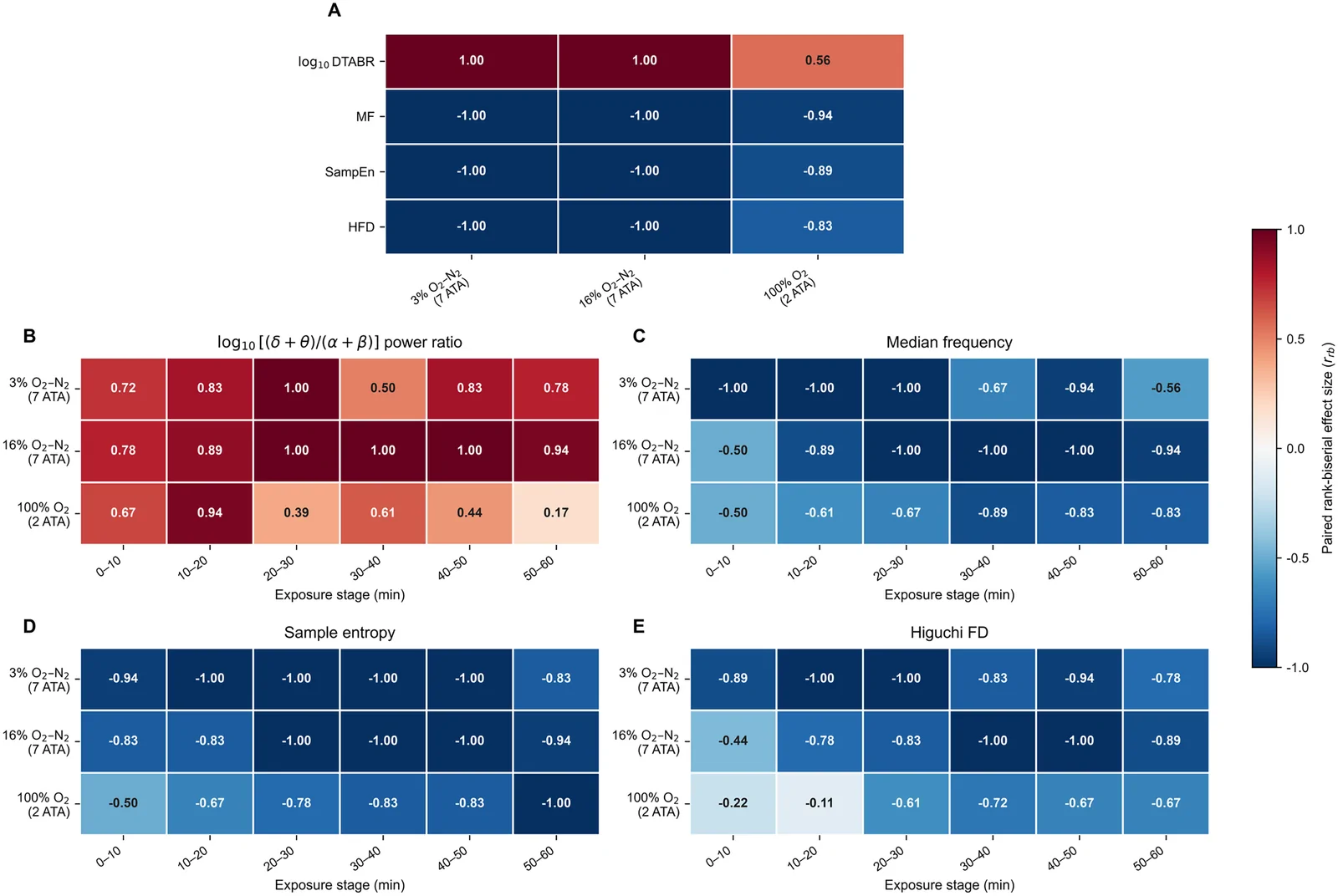
Direction of changes in principal ECoG features in the different groups. **(A)** shows the overall comparison among the three groups; **(B–E)** show the direction of changes in DTABR, MF, SampEn, and HFD, respectively, across the exposure phases. Red indicates an increase and blue indicates a decrease; the numbers within the cells represent the corresponding *r_rb_* values; n = 8 per group.

### 3.2 ECoG changes during heliox exposure

#### 3.2.1 Changes in ECoG spectral structure before and on hyperbaric heliox exposure

Compared with baseline, hyperbaric heliox exposure showed increased slow-wave activity and ECoG amplitude, with waveform fluctuations observed throughout exposure (**Figure 7A**). Spectral power was predominantly concentrated within the 1–10 Hz range and fluctuated throughout exposure, with transient increases at isolated time points (**Figure 7B**). PSD curves showed that power within the 1–20 Hz remained above baseline level during hyperbaric heliox exposure, with the increase mainly distributed within the 1–10 Hz, whereas power at frequencies above 20 Hz progressively declined below baseline level. (**Figure 7C**).

**Figure 7:**
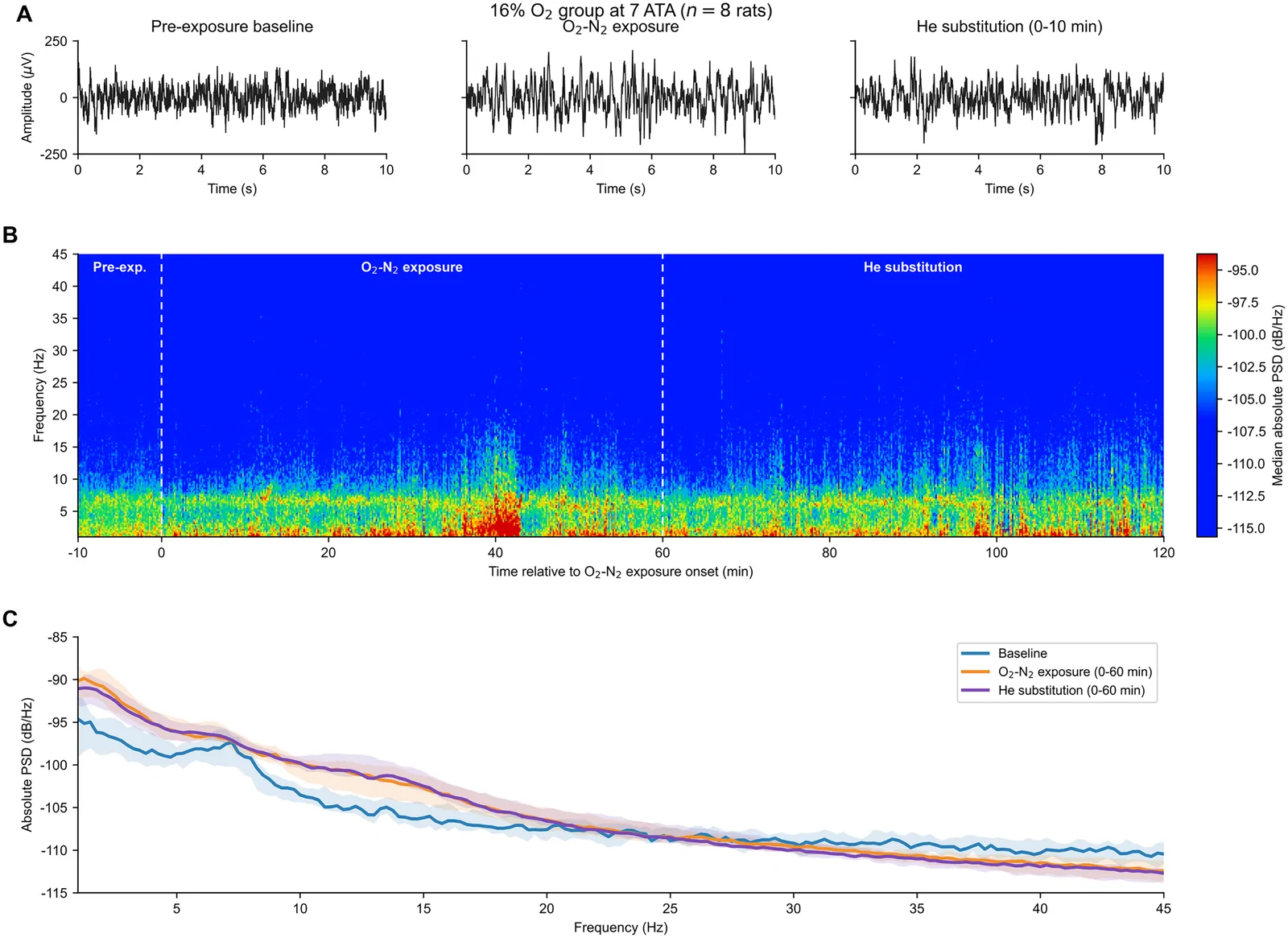
Representative ECoG waveforms, time–frequency distributions, and power spectral density on hyperbaric heliox exposure. **(A)** Representative 10-s ECoG waveforms during the baseline, hyperbaric nitrox exposure, and hyperbaric helium exposure periods. **(B)** Time–frequency maps of absolute power spectral density during baseline, hyperbaric nitrox exposure, and hyperbaric helium exposure; color represents the median across animals at each time–frequency point, and the white dashed lines indicate the onset of nitrogen exposure and helium exposure. **(C)** Power spectral density curves during baseline, hyperbaric nitrox exposure, and hyperbaric helium exposure; solid lines represent the median, and shaded areas represent Q1–Q3. For each animal, PSD was calculated for the corresponding six phases during hyperbaric nitrox exposure and hyperbaric helium exposure, and the group median and Q1–Q3 were then derived. n = 8. For clarity, only the 1–45 Hz range is shown in the time–frequency maps and power spectral density plots.

Compared with hyperbaric nitrox exposure, the range of ECoG amplitude fluctuations was narrower during hyperbaric heliox exposure (**Figure 7A**). During both exposure conditions, spectral power was predominantly concentrated within the 1–10 Hz range. However, during hyperbaric nitrox exposure, low-frequency power exhibited phase-dependent fluctuations, with increased power observed at approximately 38–43 min and extending into higher frequencies. In contrast, during hyperbaric heliox exposure, power increases were mainly confined to the low-frequency range (**Figure 7B**). PSD curves showed that power within the 1–5 Hz range was slightly lower during hyperbaric heliox exposure than during hyperbaric nitrox exposure. Within the 10–20 Hz range, power differed only slightly between the two conditions and remained above baseline levels under both conditions. At frequencies above 20 Hz, power was similar between the two conditions and remained below baseline levels (**Figure 7C**).

#### 3.2.2 Changes in absolute frequency-band power during hyperbaric nitrox exposure and subsequent heliox exposure

Compared with the baseline level, the absolute δ-, θ-, α-, and β-band power generally increased during hyperbaric heliox exposure, whereas γ-band power decreased. The median change in δ-band power ranged from 0.96 to 3.96 dB across exposure phases. The median changes in θ-, α-, and β-band power ranged from 0.29 to 3.00 dB, 1.83 to 4.00 dB, and 0.27 to 2.31 dB, respectively. γ-band power remained below baseline level across six exposure phases.

No significant overall time effects were observed for δ-band power (χ²(6) = 8.95, *P*_BH_ = 0.2944, Kendall’s W = 0.19), θ-band power (χ²(6) = 7.66, *P*_BH_ = 0.3300, Kendall’s W = 0.16), α-band power (χ²(6) = 12.16, *P*_BH_ = 0.1462, Kendall’s W = 0.25), β-band power (χ²(6) = 6.80, *P*_BH_ = 0.3394, Kendall’s W = 0.14) and γ-band power (χ²(6) = 15.43, *P*_BH_ = 0.0859, Kendall’s W = 0.32). Phase-specific comparisons showed that δ-, θ-, and α-band power at 30–40 min was significantly higher than baseline level (all *P*_BH_ = 0.0469), whereas β-band power at any exposure phase was comparable to the baseline level. γ-band power was significantly lower than baseline level across all six exposure phases (P_BH_ = 0.0234, 0.0188, 0.0188, 0.0188, 0.0188 and 0.0188, respectively) (**Figure 8B**).

**Figure 8:**
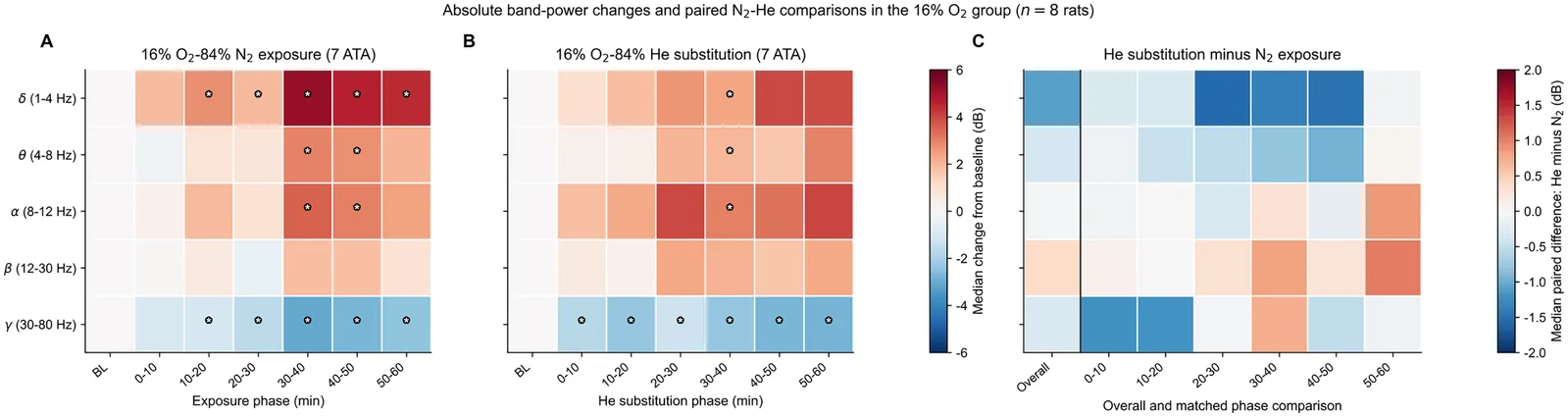
Changes in absolute frequency-band power before and after helium substitution. **(A, B)** show baseline-normalized changes in absolute frequency-band power during hyperbaric nitrox and hyperbaric heliox exposure, respectively; colors represent changes relative to each animal’s original baseline at each phase, expressed in dB, with red indicating an increase and blue indicating a decrease. **(C)** shows the overall and phase-specific paired comparisons between hyperbaric nitrox and hyperbaric heliox exposure after baseline normalization; “Overall,” represents the paired comparison between mean values across all nitrox-exposure phases and all heliox-exposure phases for each rat. The remaining six columns represent paired comparisons between nitrox- and heliox-exposure periods at the corresponding exposure phases. Red indicates higher values during heliox exposure as compared to nitrox exposure, whereas blue indicates lower values during heliox exposure. *P_BH_ < 0.05 after Benjamini–Hochberg adjustment. BL, baseline; n = 8.

Compared with hyperbaric nitrox exposure, the M (Q1, Q3) of δ-, θ-, α-, β-, and γ-band power during hyperbaric heliox exposure was −1.08 (−1.44, 0.61) dB, −0.33 (−0.92, 0.00) dB, −0.03 (−0.35, 0.40) dB, 0.37 (0.20, 0.62) dB and −0.31 (−1.01, 0.22) dB, respectively, with no significant differences (*P*_BH_ = 0.1953–0.9453).

Phase-specific comparisons showed that δ-band power was lower during all six phases of hyperbaric heliox exposure than that during hyperbaric nitrox exposure, and the median difference in δ-band power ranged from −1.56 to −0.07 dB between two conditions; β-band power was generally higher during heliox exposure, except at isolated phases, and the median difference in β-band power ranged from −0.01 to 1.02 dB. θ-, α-, and γ-band power showed phase-dependent increases or decreases, and the median differences ranged from −0.95 to 0.06 dB, −0.32 to 0.86 dB, and −1.21 to 0.73 dB, respectively, between two conditions. No significant differences in α-, β-, θ-, γ-, or δ-band power were observed between two exposure conditions at any phase (*P*_BH_ = 0.1406–1.0000) (**Figure 8C**).

#### 3.2.3 Changes in principal ECoG features before and during hyperbaric heliox exposure

Compared with baseline, none of four principal ECoG features showed a significant changes over time during hyperbaric heliox exposure: DTABR (χ²(6) = 5.04, *P*_BH_ = 0.5392, Kendall’s W = 0.10), MF (χ²(6) = 7.66, *P*_BH_ = 0.3520, W = 0.16), SampEn (χ²(6) = 15.64, *P*_BH_ = 0.0632, W = 0.33) and HFD (χ²(6) = 13.29, *P*_BH_ = 0.0774, W = 0.28). Phase-specific comparisons showed that the median paired differences in DTABR during hyperbaric heliox exposure ranged from −0.031 to 0.084, with no significant differences at any phase as compared to the baseline level (*P*_BH_ = 0.5000–0.9453). MF was lower than baseline level at all phases, with median paired differences ranging from −1.93 to −1.01 Hz, although phase-specific comparisons showed no statistical significance (*P*_BH_ = 0.0703–0.1953). SampEn was significantly lower than the baseline level at all six phases (*P*_BH_ = 0.0391, 0.0234, 0.0391, 0.0156, 0.0156 and 0.0156, respectively), with median paired differences ranging from −0.51 to −0.25. HFD was significantly lower than the baseline level at all six phases (*P*_BH_ = 0.0391, 0.0234, 0.0391, 0.0234, 0.0234, and 0.0234, respectively), with median paired differences ranging from −0.218 to −0.107 (**Figures 9A–D**).

**Figure 9:**
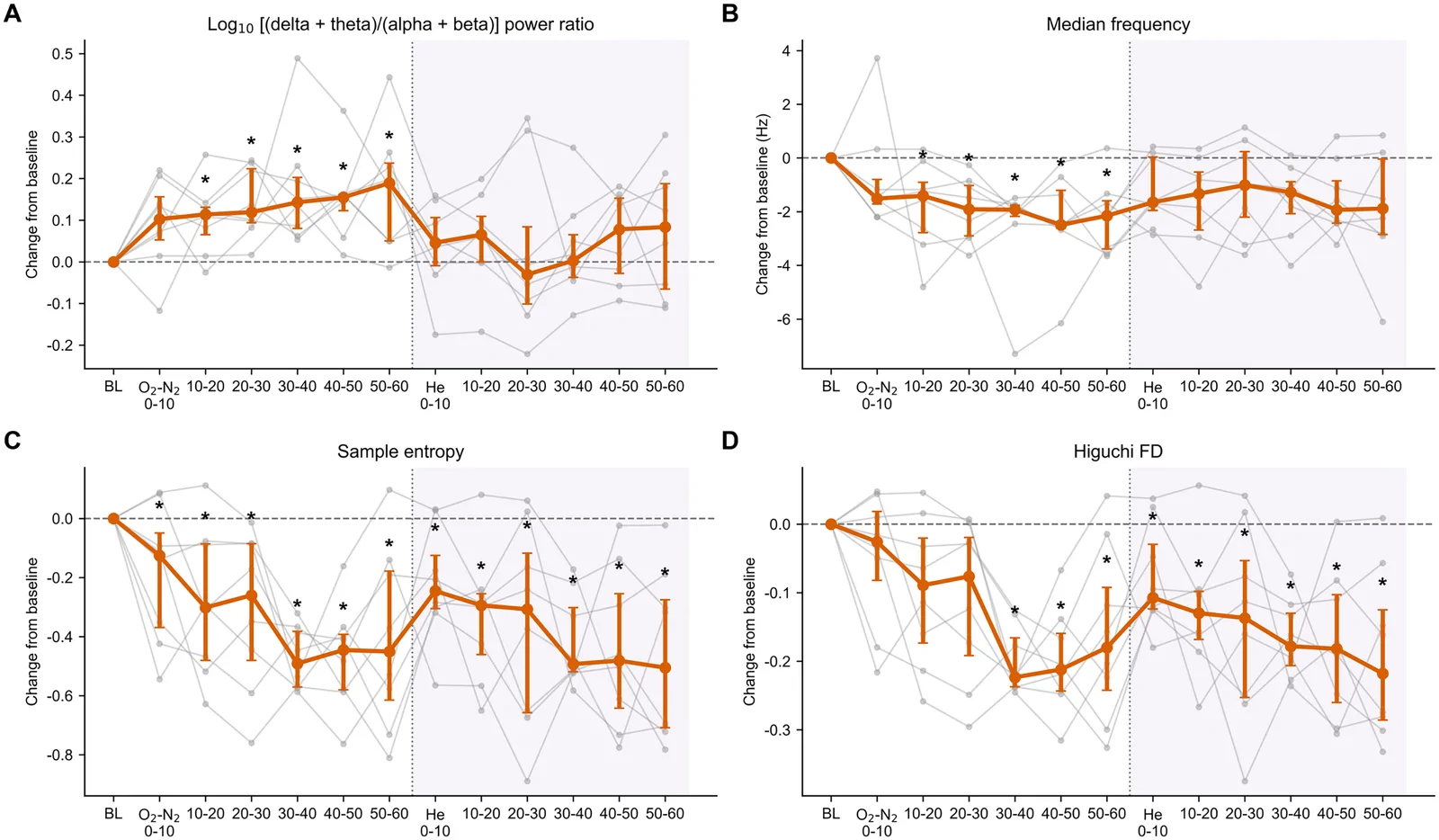
Dynamic changes in principal ECoG features during hyperbaric nitrox exposure and subsequent hyperbaric helium exposure. **(A–D)** show DTABR, MF, SampEn, and HFD, respectively. The vertical dashed line indicates the transition from hyperbaric nitrox to hyperbaric helium, and the light purple background denotes the helium substitution phase. Thin gray lines represent changes in individual rats, whereas the thick orange line and error bars represent the group median and the first and third quartiles. The y-axis represents the change at each phase relative to baseline, with positive values indicating values above baseline and negative values indicating values below baseline. BL, baseline; n = 8. *P_BH_ < 0.05 vs. the original baseline.

Exploratory comparisons were performed between hyperbaric heliox exposure phases and 30–40 min phase of hyperbaric nitrox exposure. For each rat, the mean value across six hyperbaric heliox exposure phases was first calculated and then compared with that at 30–40 min of hyperbaric nitrox exposure. During hyperbaric heliox exposure, the M (Q1, Q3) of DTABR, MF, SampEn, and HFD was −0.095 (−0.189, −0.003), 1.01 (0.24, 1.86) Hz, 0.163 (−0.010, 0.245), and 0.075 (0.010, 0.114), respectively, with no significant differences for four features (all *P*_BH_ = 0.2500). Phase-specific comparisons showed that DTABR was lower at all phases of hyperbaric heliox exposure than that at 30–40 min of hyperbaric nitrox exposure, with median differences ranging from −0.156 to −0.067 (*P*_BH_ = 0.2344–0.2500). MF was higher at all heliox-exposure phases, with median differences ranging from 0.42 to 1.62 Hz (*P*_BH_ = 0.2930–0.3125). The median differences ranged from −0.074 to 0.265 for SampEn (*P*_BH_ = 0.2344–1.0000) and from −0.018 to 0.121 for HFD (*P*_BH_ = 0.2344–0.8438). None of these parameters differed significantly between any phase of hyperbaric heliox exposure and the 30–40 min phase of hyperbaric nitrox exposure (**Figures 10A–D**).

**Figure 10:**
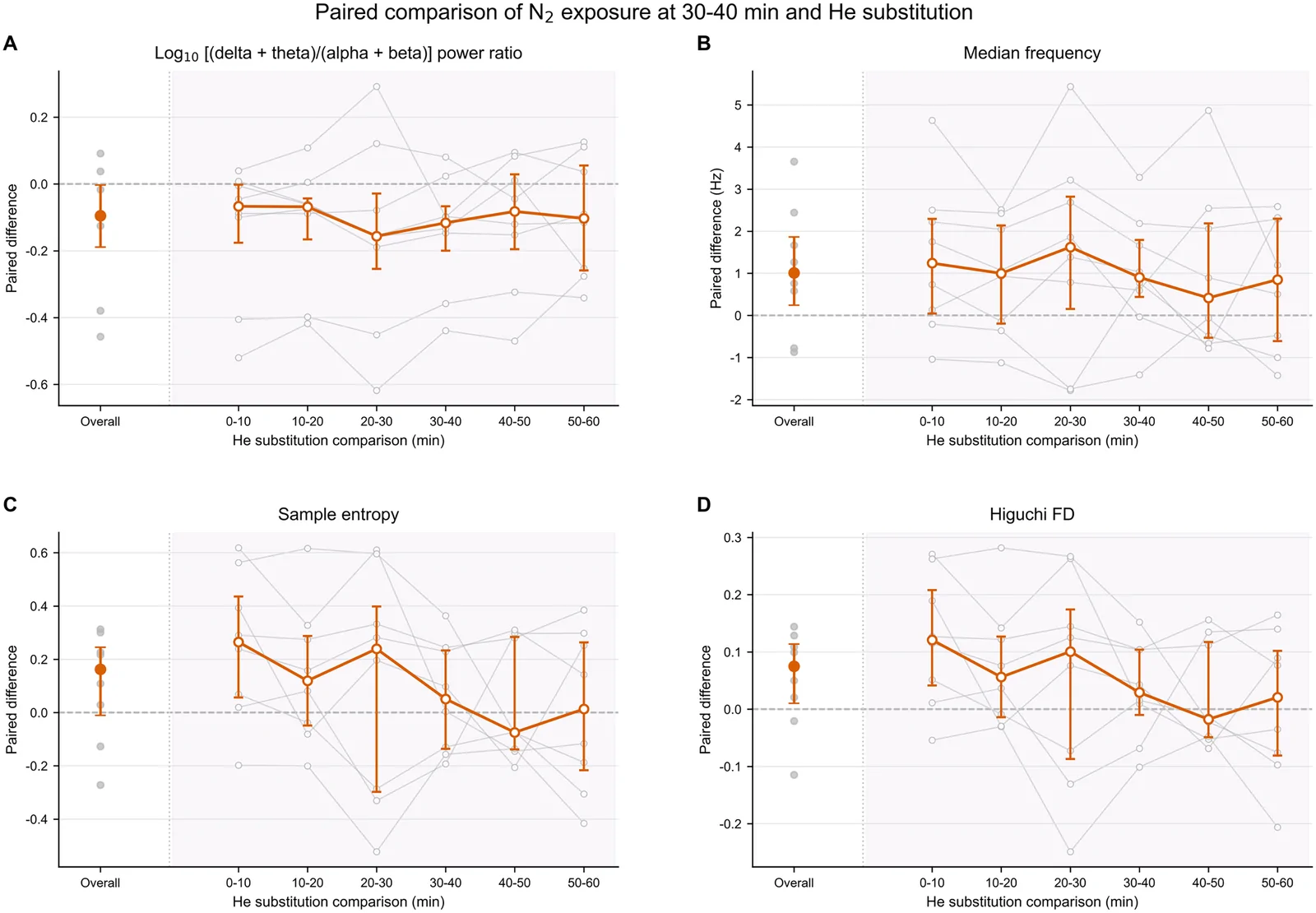
Principal ECoG features during hyperbaric heliox exposure relative to those at 30–40 min of hyperbaric nitrox exposure. **(A–D)** show DTABR, MF, SampEn, and HFD, respectively. Using the 30–40 min phase of hyperbaric nitrox exposure as the reference, the y-axis shows paired differences, with positive values indicating higher values during hyperbaric heliox exposure and negative values indicating lower values. “Overall” represents the mean value across six phases of hyperbaric heliox exposure for each rat. Gray dots and thin lines represent individual rats, whereas orange dots and error bars represent the median and the first and third quartiles (n = 8). *P_BH_ < 0.05 *vs*. 30–40 min phase of hyperbaric nitrox exposure.

#### 3.2.4 Direction of ECoG changes during hyperbaric nitrox exposure and subsequent heliox exposure

Relative to baseline, the overall comparison during hyperbaric heliox exposure showed that DTABR increased in 6/8 rats (*r_rb_* = 0.50), whereas MF decreased in 7/8 rats (*r_rb_* = -0.89), SampEn and HFD decreased in 8/8 rats (*r_rb_* = -1.00) (**Figure 11**). Phase-specific*r_rb_* values ranged from −0.06 to 0.56 for DTABR, −0.94 to −0.56 for MF, −1.00 to −0.83 for SampEn, and −1.00 to −0.83 for HFD. Across six exposure phases, DTABR was above baseline in 5/8, 6/8, 3/8, 4/8, 5/8, and 5/8 rats, respectively; MF was below baseline level in 6/8, 6/8, 5/8, 7/8, 7/8, and 6/8 rats, respectively; SampEn was below baseline level in 6/8, 7/8, 6/8, 8/8, 8/8, and 8/8 rats, respectively; and HFD was below baseline level in 6/8, 7/8, 6/8, 8/8, 7/8, and 7/8 rats, respectively (**Figure 11**).

**Figure 11:**
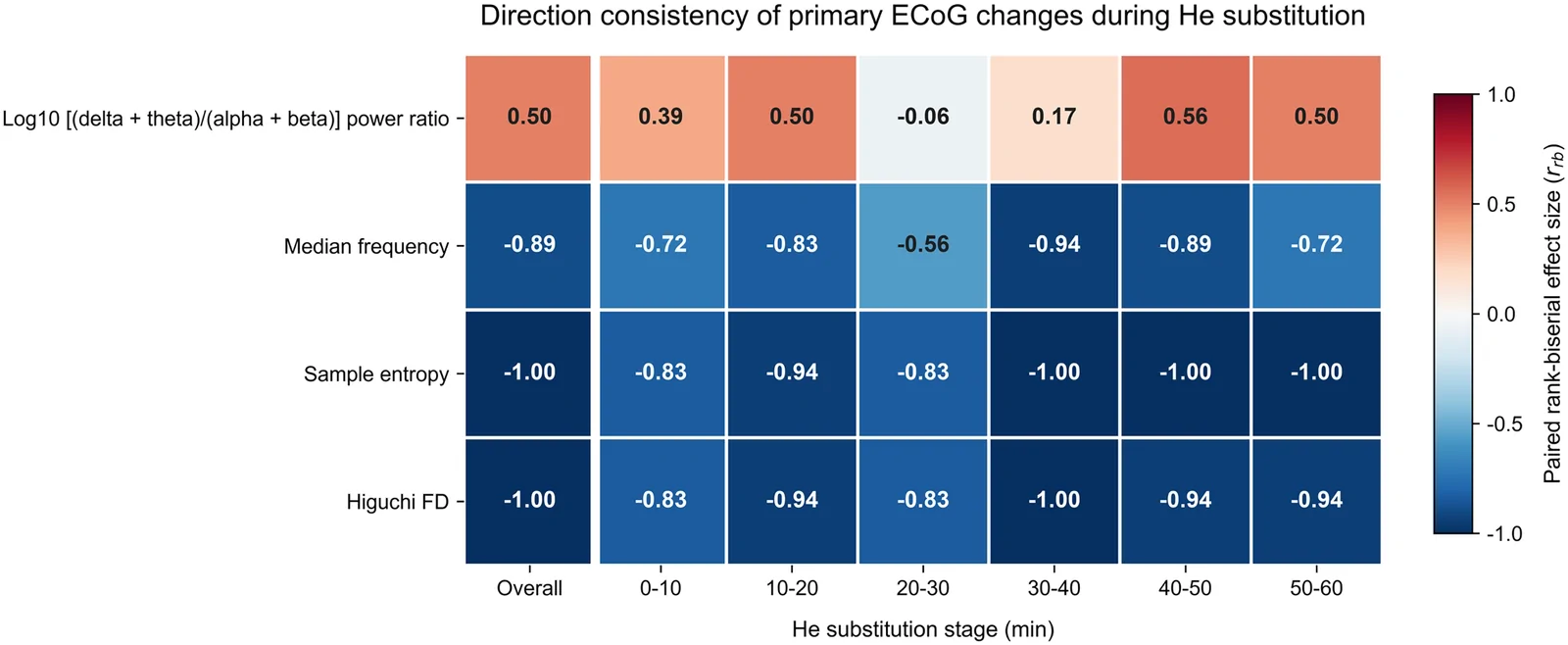
Consistency in the direction of baseline-relative changes in principal ECoG features during hyperbaric helium exposure. The heat map shows *r_rb_* for overall and phase-specific changes relative to the baseline level during hyperbaric heliox exposure (n = 8). “Overall” represents the comparison between mean across six phases of hyperbaric heliox exposure and baseline for each rat; red indicates an increase, blue indicates a decrease, and the numbers within the cells represent *r_rb_*.

Using the 30–40 min phase of hyperbaric nitrox exposure as the reference, the overall comparison showed that DTABR decreased in 6/8 rats (*r_rb_* = -0.72), whereas MF increased in 6/8 rats *r_rb_* = 0.61), SampEn increased in 6/8 rats (*r_rb_* = 0.50), and HFD increased in 6/8 rats (*r_rb_* = 0.56) (**Figure 12**). Phase-specific *r_rb_* values ranged from −0.83 to −0.50 for DTABR, 0.44 to 0.67 for MF, 0 to 0.83 for SampEn, and 0.11 to 0.83 for HFD. Across six hyperbaric heliox exposure phases, DTABR was lower than the reference in 6/8, 6/8, 6/8, 6/8, 5/8, and 5/8 rats, respectively; MF was higher in 6/8, 5/8, 6/8, 6/8, 4/8, and 5/8 rats, respectively; SampEn was higher in 7/8, 5/8, 5/8, 5/8, 3/8, and 4/8 rats, respectively; and HFD was higher in 7/8, 5/8, 5/8, 6/8, 3/8, and 4/8 rats, respectively (**Figure 12**).

**Figure 12:**
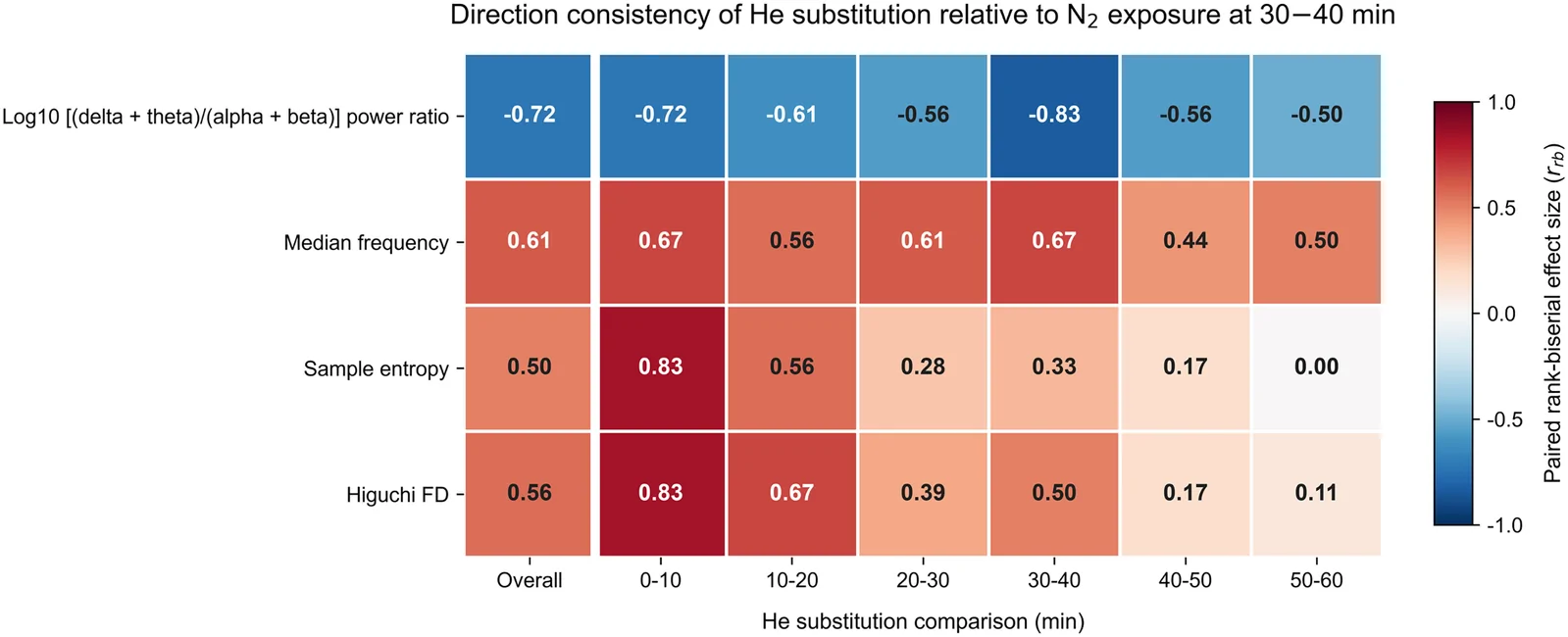
Direction and consistency of changes in principal ECoG features during hyperbaric heliox exposure relative to those at 30–40 min of hyperbaric nitrox exposure. Using the 30–40 min phase of hyperbaric nitrox exposure as the reference, the heat map shows the *r_rb_* for overall and phase-specific comparisons during hyperbaric heliox exposure relative to this reference (n = 8). “Overall” represents the comparison between mean across six phases of hyperbaric heliox exposure for each rat and the 30–40 min phase of hyperbaric nitrox exposure. Red indicates an increase, blue indicates a decrease, and the numbers within the cells represent *r_rb_* values.

## 4 Discussion

This study characterized the temporal evolution of rat ECoG activity during hyperbaric nitrox exposure and subsequent hyperbaric heliox exposure. Both 16% O_2_–N_2_ and 3% O_2_–N_2_ groups exhibited enhanced slow-wave activity and a shift in spectral power toward lower frequencies, accompanied by reduced MF, SampEn, and HFD, increased DTABR, and decreased γ-band power during several exposure phases. These changes generally appeared earlier in the 3% O_2_–N_2_ group than in the 16% O_2_–N_2_ group. In contrast, the 100% O_2_ group exhibited relatively modest changes in ECoG measures. The direction of these changes was highly consistent among most animals in both nitrox groups, suggesting a consistent pattern of cortical electrophysiological alterations under the tested exposure conditions. Increased δ- and θ-band activity, together with reduced MF and elevated DTABR, indicated a shift in the spectral distribution of ECoG activity toward lower frequencies. Concurrent reductions in SampEn and HFD suggested greater temporal regularity and reduced waveform complexity, respectively, providing complementary descriptions of changes in the underlying ECoG signal. The accompanying decrease in γ-band power further suggested attenuation of higher-frequency cortical activity. Collectively, these findings suggest that hyperbaric nitrox exposure is associated with a shift toward slower, more regular, and less complex cortical ECoG patterns, potentially reflecting reduced fast cortical network activity.

Previous studies have reported shifts in EEG activity toward lower frequencies during hyperbaric exposure to nitrogen-containing mixture. Pastena et al. (2005) reported marked increases in δ- and θ-band activity in humans breathing hyperbaric air at 900 kPa, with these changes evolving over the course of exposure. Peng et al. (2019) found that mice exposed to hyperbaric nitrox exhibited a marked reduction in mean EEG frequency, accompanied by decreased fast-wave activity and increased slow-wave activity. These findings are consistent with the enhanced slow-wave activity and reduced MF observed in the present study. However, Kinney et al. (1977) found no significant changes in α- or θ-band activity in humans exposed to hyperbaric air at 700 kPa, despite the presence of abnormalities in visual evoked potentials. The PN_2_ under their experimental conditions was approximately 547–553 kPa, lower than the 679 kPa and 588 kPa achieved in the 3% O_2_–N_2_ and 16% O_2_–N_2_ groups, respectively, in the present study. Differences in PN_2_ may therefore have contributed to the variability in electrophysiological responses across studies. In addition, Kinney et al. used human scalp EEG, whereas the present study employed continuous invasive ECoG recording, which reduces signal attenuation by the scalp and skull and facilitates temporal monitoring of cortical electrical activity throughout exposure. Combined with spectral and nonlinear signal analyses, this approach may help characterize temporal alterations that are not fully captured by conventional frequency-band power measures alone.

The sustained reductions in SampEn and HFD observed during hyperbaric nitrox exposure in the present study extend previous findings on electrophysiological alterations associated with elevated nitrogen partial pressure. Because both metrics were derived from the same ECoG signal, their reductions should be interpreted as complementary features of an overall electrophysiological change rather than fully independent evidence of reduced neural complexity. Previous studies of anesthetic states have shown that reduced levels of consciousness are often accompanied by decreased EEG complexity, reflecting a shift toward more regular and less differentiated patterns of neural activity. The electrophysiological changes observed in the present study resemble this anesthesia-associated pattern; however, this similarity does not establish that hyperbaric nitrox exposure and pharmacological anesthesia share identical underlying mechanisms. Peng et al. (2018, 2019) reported that hyperbaric nitrox exposure could suppress NR2B-containing NMDA receptor activity and downstream CREB signaling, potentially contributing to the observed slowing of ECoG activity and reduction in signal complexity. However, because neurotransmitter and receptor alterations were not directly assessed in the present study, this proposed mechanism requires further experimental validation.

Rats in the 3% O_2_–N_2_ and 16% O_2_–N_2_ groups were exposed at 700 kPa, during which the nitrogen partial pressures was 679 kPa and 588 kPa, respectively and the oxygen partial pressures was 21 kPa and 112 kPa, respectively. Given the substantially higher PO_2_ in the 16% O_2_–N_2_ group, a 100% O_2_ group was included to assess the contribution of elevated oxygen partial pressure to the observed ECoG changes. ECoG activity was continuously recorded and analyzed throughout exposure. In the 100% O_2_ group, rats were exposed to pure oxygen at 200 kPa. The pressure in the 100% O_2_ group was no higher than 200 kPa, aiming to reduce the risk of acute central nervous system oxygen toxicity. ECoG alterations during pure oxygen exposure were markedly smaller than those observed in the 3% O_2_–N_2_ and 16% O_2_–N_2_ groups, suggesting that elevated PO_2_ alone may not fully account for the observed changes during hyperbaric nitrox exposure. However, because rats in the 100% O_2_ group were exposed to pure oxygen at 200 kPa and those in two nitrox groups were exposed to nitrox at 700 kPa, the contribution of total ambient pressure could not be isolated. These findings may therefore reflect the combined influences of elevated nitrogen partial pressure and other aspects of hyperbaric exposure. ECoG changes also appeared earlier in the 3% O_2_–N_2_ group than in the 16% O_2_–N_2_ group, which may be related to the higher nitrogen partial pressure in the former, although the intergroup difference in oxygen partial pressure should also be considered.

To mitigate the narcotic effects associated with elevated nitrogen partial pressure, helium, an inert gas with lower narcotic potency, is commonly used in breathing mixtures for deep diving. To further examine ECoG changes following hyperbaric heliox exposure, the nitrox in the 16% O_2_–N_2_ group was replaced with heliox at the same pressure, and ECoG activity was continuously monitored. Following helium substitution, DTABR tended to decrease, whereas MF, SampEn, and HFD tended to increase, collectively showing a trend toward baseline levels. Vrijdag et al. (2022) reported alterations in EEG functional connectivity during hyperbaric air exposure in humans, whereas comparable changes were not observed during heliox exposure at the same pressure. When compared with the 30–40 min stage of hyperbaric nitrox exposure, ECoG measures during subsequent heliox exposure generally returned toward baseline; however, neither the overall analyses nor the stage-specific paired comparisons of DTABR, MF, SampEn, and HFD reached statistical significance. Moreover, some measures remained below their pre-exposure baseline levels during hyperbaric heliox exposure. These findings suggest the ECoG activity showed a shift toward baseline following helium substitution but the electrophysiological measures did not completely return to baseline levels. The observed pattern may be explained as the short time required for tissue nitrogen washout, the gas-exchange process, and the fixed experimental sequence. Moreover, the relatively small sample size may also limit the ability to detect statistically significant differences.

Several limitations of this study should be acknowledged. First, the sample size was relatively small, with eight animals per group, and inter-individual variability in ECoG responses under the same exposure conditions may have influenced the results. Second, ECoG recording was limited to a single differential channel, whereas multichannel EEG is commonly used in clinical settings. Therefore, caution is warranted when extrapolating these findings to humans. As noted above, nitrogen narcosis in humans is assessed primarily through behavioral, cognitive, and affective manifestations. However, corresponding changes in affect and motor coordination are more difficult to assess reliably in animals, particularly without predefined quantitative behavioral assessments, limiting the ability to validate the observed ECoG alterations against neurobehavioral outcomes. Further studies with rigorous experimental designs are needed to characterize ECoG alterations during hyperbaric nitrox exposure and clarify their relationship with neurobehavioral changes.

## 5 Conclusion

This study identified marked alterations in rat ECoG activity during hyperbaric nitrox exposure, characterized primarily by enhanced low-frequency activity, a shift in spectral distribution toward lower frequencies, and reduced signal complexity. These findings indicate a shift in cortical electrophysiological activity toward slower and more regular patterns under the tested exposure conditions. In addition, the higher the nitrogen partial pressure, the earlier these changes emerged. ECoG changes during hyperbaric oxygen exposure were relatively small; however, differences in oxygen partial pressure and total ambient pressure across groups preclude attributing the observed alterations exclusively to nitrogen partial pressure.

Following helium substitution, DTABR, MF, SampEn, and HFD generally showed trends toward baseline levels, although the corresponding comparisons did not reach statistical significance. Therefore, the present findings do not establish that helium substitution reverses the electrophysiological alterations associated with hyperbaric nitrox exposure. Overall, the identified ECoG features characterize cortical electrophysiological changes during hyperbaric nitrox exposure and may serve as candidate measures for future investigations into their relationship with nitrogen narcosis. Further studies incorporating larger sample sizes, multichannel electrophysiological recordings, and integrated behavioral and neurological assessments are warranted to validate these electrophysiological features and clarify their relationship with the severity of nitrogen narcosis.

## Data Availability

The raw data supporting the conclusions of this article will be made available by the authors, without undue reservation.
