# Supplementary material for: Electrocorticographic characteristics of rats exposed to hyperbaric nitrox
Source: Front Physiol. 2026 Oct 21;17:1976565. doi: 10.3389/fphys.2026.1976565 (PMC13730938; doi:10.3389/fphys.2026.1976565)
Supplement: Supplementary file 1 [file Table1.docx]

Supplementary Methods：

Each animal was treated as an independent experimental unit, and individual 10-s epochs were not considered independent statistical observations. Each group comprised eight animals. Given the relatively small sample size and the potentially different distributional characteristics of the ECoG-derived measures, no assumption of normality was made for continuous variables, and two-sided rank-based nonparametric methods were prespecified for statistical inference. Continuous variables across animals are presented as the median (first quartile, third quartile), denoted as M (Q1, Q3). P values arising from the relevant comparisons were adjusted within the prespecified families described below using the Benjamini–Hochberg (BH) procedure; adjusted P values are denoted as *P*_BH_, with *P*_BH_ < 0.05 considered statistically significant.

Within each experimental group, overall differences between baseline and the six exposure phases were assessed using the Friedman test, with Kendall’s W reported as the effect size. P values from the Friedman tests for frequency-band power measures and other ECoG metrics were adjusted separately using the Benjamini–Hochberg (BH) procedure.

Each exposure phase was compared with the corresponding baseline using the paired Wilcoxon signed-rank test. For each metric within each experimental group, the six P values obtained from comparisons between the six exposure phases and baseline were adjusted together using the Benjamini–Hochberg (BH) procedure. A directional paired rank-biserial correlation coefficient t$r_{rb}$ was also calculated, with the direction defined as “exposure-phase value minus baseline value”; positive values indicate values above baseline, whereas negative values indicate values below baseline.

Before the overall comparison among the three groups, each metric was transformed into a change relative to each animal’s own baseline. For each animal, the arithmetic mean across the six exposure phases was first calculated, and the resulting animal-level values were then compared among the three groups using the Kruskal–Wallis test. The five P values for the five frequency-band power measures were adjusted together using the BH procedure, whereas the four P values for the four principal ECoG features were adjusted as a separate family. When the overall three-group comparison reached statistical significance, pairwise comparisons were performed using the Mann–Whitney U test. For each metric, the three P values obtained from pairwise comparisons among the three experimental groups were adjusted together using the BH procedure, and the $r_{rb}$ was calculated as the effect size.

For phase-specific comparisons among the three groups, the Kruskal–Wallis test was performed separately at each exposure phase. The 30 P values generated for the five frequency bands across the six exposure phases were adjusted together using the BH procedure, whereas the 24 P values generated for the four principal ECoG features across the six phases were adjusted as a separate family. When the overall three-group comparison for a given metric at a specific exposure phase reached statistical significance, pairwise comparisons among the three groups were performed using the Mann–Whitney U test, and the three resulting P values for that metric at that phase were adjusted together using the BH procedure.

During hyperbaric helium exposure, overall differences between the original baseline and the six helium-exposure phases were assessed using the Friedman test. Each helium-exposure phase was compared with the original baseline using the paired Wilcoxon signed-rank test; for each metric, the six P values obtained from comparisons between the six helium-exposure phases and baseline were adjusted together using the Benjamini–Hochberg (BH) procedure.

For comparisons of frequency-band power between the hyperbaric nitrogen and hyperbaric helium exposure periods, the arithmetic mean across the six phases of each exposure period was first calculated for each animal, followed by an overall paired comparison using the Wilcoxon signed-rank test; the five P values obtained for the five frequency-band power measures were then adjusted together using the Benjamini–Hochberg (BH) procedure. The corresponding time phases of the two exposure periods were subsequently compared using paired tests; for each frequency-band power measure, the six P values obtained across the six phase-specific comparisons were adjusted together using the BH procedure.

For comparisons of the four principal ECoG features, the 30–40 min phase of hyperbaric nitrogen exposure was used as the reference condition. For each animal, the arithmetic mean across the six phases of hyperbaric helium exposure was first calculated and compared with the corresponding value at 30–40 min of hyperbaric nitrogen exposure using the paired Wilcoxon signed-rank test; the four P values obtained for the four ECoG features were adjusted together using the Benjamini–Hochberg (BH) procedure. Each phase of hyperbaric helium exposure was then compared separately with the 30–40 min phase of hyperbaric nitrogen exposure; for each ECoG feature, the six P values obtained from these phase-specific comparisons were adjusted together using the BH procedure. For all comparisons between hyperbaric helium exposure and the 30–40 min phase of hyperbaric nitrogen exposure, the $r_{rb}$ was calculated, with its direction defined as “helium-exposure value minus the value at 30–40 min of nitrogen exposure.”

Overall differences among the three groups in the proportions of animals showing increases versus decreases were assessed using the Fisher–Freeman–Halton exact test. When the overall comparison reached statistical significance, pairwise comparisons were performed using Fisher’s exact test, and the three resulting P values were adjusted together using the Benjamini–Hochberg (BH) procedure.
